# Supplementary material for: Gastric residual volume measurement in British neonatal intensive care units: a survey of practice
Source: BMJ Paediatr Open. 2020 Aug 7;4(1):e000601. doi: 10.1136/bmjpo-2019-000601 (PMC7418677; doi:10.1136/bmjpo-2019-000601)
Supplement: Supplementary data [file bmjpo-2019-000601supp001.pdf]

Introduction

We want to find out how Gastric Residual Volumes (GRV) are measured and used in your NICU. GRV measurement is aspirating ALL of the stomach contents to measure a 'volume' or 'aspirate' and often to look at the colour. This is NOT the small amount that is aspirated to test pH to confirm tube position.

We recommend a senior clinical nurse (who still does clinical practice) along with a senior doctor and/or a dietitian sit down together to complete this survey and we would like just one survey returned per NICU. If this is not possible, please respond without such a meeting.

Which neonatal unit do you work at?

Is the unit?

◯ NICU surgical and medical
◯ NICU medical only
◯ LNU
◯ SCBU

What are the job positions of the people completing this survey?

☐ Senior Doctor (Consultant)
☐ Middle Grade Doctor (SpR)
☐ Junior Grade Doctor (SHO)
☐ Nurse
☐ Dietitian

What band of nurse are you?

____

What band of dietitian are you?

____

Does your unit have written feeding guidelines/ protocol for medical and surgical babies?

◯ Yes
◯ No

We would greatly appreciate it if you would upload the feeding guidelines/protocol using the 'upload document' link below. Alternatively, they can be emailed to the GASTRIC study team at gastric.study@liverpool.ac.uk.

Are your standard NG feeds:

◯ Intermittent
◯ Continuous

Is there specific guidance about how Gastric Residual Volume should be measured and interpreted - for example a protocol or guideline?

◯ Yes
◯ No

We would greatly appreciate it if you would upload the guidelines/protocol using the 'upload document' link below. Alternatively, they can be emailed to the GASTRIC study team at gastric.study@liverpool.ac.uk.

Does Gastric Residual Volume measurement differ between the medical and surgical babies?

◯ Yes
◯ No

Please describe how it differs?

The rest of the survey is now ONLY asking about the management of preterm and term medical babies - not surgical babies.

How often do staff in your unit measure Gastric Residual Volume? (this question is NOT about aspirating a small amount to test pH, ONLY about how often you measure Gastric Residual Volume)

☐ Once a day
☐ Before every feed
☐ Only when clinically indicated
☐ Other

Please provide further details regarding how staff measure Gastric Residual Volume below; if 'other' has been selected, please also describe below:

Are Gastric Residual Volume measured for all babies, or just below a set gestational age/birth weight or for a specific condition?

Is the specific guidance for Gastric Residual Volume measurement followed and actually undertaken as per protocol? If needed, please ask a clinical member of your nursing team.

○ Always
○ Usually
○ Often
○ Rarely
○ Never

Why is the guidance not always followed?

Who usually decides what to do with concerning Gastric Residual Volume aspirates in the first instance?

☐ Senior Doctor (Consultant)
☐ Middle Grade Doctor (SpR)
☐ Junior Grade Doctor (SHO)
☐ Bedside Nurse
☐ Nurse in charge of shift (senior nurse)

What band of nurse?

What would make a nurse seek advice from the medical team?

If you indicated that more than one person is involved in decision-making, please explain which factors may influence who makes the decision below:

How much does volume of the aspirate affect your decision around Gastric Residual Volume?

◯ 1 (Not at all) ◯ 2 ◯ 3
◯ 4 ◯ 5 (Very much)

Please specify how:

How much does colour of the aspirate affect your decision around Gastric Residual Volume?

◯ 1 (Not at all) ◯ 2 ◯ 3
◯ 4 ◯ 5 (Very much)

Please specify how:

What do you do with Gastric Residual Volume once obtained: return it or discard it?

◯ Return
◯ Discard
◯ Other

Please explain the factors that influence the decision to return or discard the Gastric Residual Volume. If 'other' selected, please also explain below:

Thank you for participating in the survey, your responses are greatly appreciated! If you have any further comments you wish to make, please provide below:

If you wish to be contacted about the study, please add your email address below (optional):

____
