# Supplementary material for: Gastric residual volume measurement in British neonatal intensive care units: a survey of practice
Source: BMJ Paediatr Open. 2020 Aug 7;4(1):e000601. doi: 10.1136/bmjpo-2019-000601 (PMC7418677; doi:10.1136/bmjpo-2019-000601)
Supplement: Supplementary data [file bmjpo-2019-000601supp002.pdf]

Supplementary Table 1: Individual responses to the question "Are Gastric Residual Volume measured for all babies, or just below a set gestational age/birth weight or for a specific condition"?

	Frequency
<32/40	2
All Babies	**30**
All babies on high risk feeding regimes	1
All babies until on full feeds	1
All babies weighing <1500g at birth who have not reached full feeds	1
All babies with NG tubes in until decision to stop monitoring ie for long term tube feeding.	1
All babies with NG tubes.	1
All babies with NGT/ OGT in-situ	1
All babies with tube feeding whilst the feeds are being increased	1
All tube fed babies	1
Any baby with a gastric tube in situ - but again, this is very variable depending on the nurse looking after the baby	1
As clinically indicated	1
Babies at high/moderate risk [of NEC] on our enteral feeding guideline - <32 weeks	1
Currently for all babies but stopping soon unless unwell.	1
For all babies on NG tube feeds	1
For premature babies or for sick babies on iv fluids and hourly feeds. NIC not aware of gestation cut off.	1
For specific conditions	1
GRV will be measured for babies who are on an increasing feed volume, to assess how well the baby is absorbing feed and to determine if the baby will be able to tolerate an increase in their milk volume.	1
GRVs are routinely measured 4-6 hourly for all babies	1
If clinically indicated. No set policy.	1
If requested by medical team	1
In intensive care - yes or in babies who are grading up to full enteral feeds	1
It would depend on the clinical situation	1
Measure for all babies when commencing enteral NGT feeds	1
NO	1
No baby with only medical issues; will depend on surgical condition/ stage pre and post surgery in surgical babies	1
No set criteria.	1
No set cut-offs, individual nurses make decision to measure or not	1
No. Only if showing signs/symptoms of not tolerating feeds.	1
No. babies within ICU may have GRV measured when establishing feed.	1
Not routinely done	1
Only if clinically indicated or if on continuous feeds (used in smallest babies below <34 weeks)	1
Only those being fed more frequently than 3 hourly - no specific cut-off but in general <32 weeks or IUGR or concern about antenatal Dopplers	1
Only when clinically indicated in any baby	1
Only when clinically indicated.	1
Our practice is not to empty out the stomach completely to measure residual volume. If a significant volume is aspirated prior to the next feed(~50% of the feed volume), then the feed is replaced unless there is concern regarding the nature of the aspirate or the clinical condition of the baby.	1

The nature of the aspirate is often commented on. In an observational study, green stained aspirates were of themselves not a marker of feed intolerance if of small volume (<2mL for infants <750g, or <3mL for infants <1kg). However, Advancing enteral feeds Weight <1001g 1001-1250g 1251-1500g or <33/40 Stage 2 Initial Nutritive Feeds 30ml/kg/day 30ml/kg/day 30ml/kg/day Stage 3 24 Hourly Increments 30ml/kg/day 30ml/kg/day 30ml/kg/day a dark stained gastric aspirate is generally accepted as abnormal when a feeding tube is believed to be correctly positioned in the stomach. An appropriate GRV is <25% of preceding volume administered since last replacement of gastric aspirate. In this case, the aspirated volume should be replaced in full. A volume of GRV >25% but less than 1.5mL is unlikely to be of concern and can again be replaced. A GRV of 25-50% of given volume is relatively high. If the infant is well and there is no clinical evidence of NEC, then the normal hourly volume should be replaced only. Feeds may be continued, but there should be no increments in feed until GRV has been below 25% on two consecutive occasions. A GRV of greater than 50% of preceding volume administered is excessive and should prompt a clinical assessment. If the examination is unremarkable and the infant is well, the hourly amount should be replaced and the feed omitted. If the following aspirate before the next scheduled feed has reduced then feeds can be continued, but sustained aspirates of >50% should prompt further review and withholding feeds for 12 to 24 hours even when clinical assessment is normal. On restarting feeds, it is recommended to recommence feeds at 10ml/kg/d for 6 hours, then 50% of previously attained feeds for 6 hours then back to previously attained feed rate. No further increments should be attempted for a further 24 hours. Lightly bile stained aspirates can be tolerated if of accept See attached feeding guideline as above	1
This is not stated precisely in our guidelines. Anecdotally the smaller more immature babies and the surgical babies get them measured more frequently	1
We always check the tube before a feed is given but we only measure GRV if indicated and this would be in all babies If when checking the tube the nurse finds it easy to withdraw milk then she will often check there isn't a large residual volume.	1
We would normally not measure gastric residual volume before ngt feeding a baby as used to be done in the past. A small amount to test pH is all that is done here.	1
Yes	1
all babies being NGT fed	1
all babies if ng tube in place	1
all babies on NGT feeds	1
all babies until fully fed once on full enteral feeds will just check pH	1
all tube fed	1
as above	1
measured for all as clinically indicated.	1
most/all	1
n/a	1
no	2
no set gestation (all NG fed babies)	1
no specific gestational age.	1
not routinely measured	1
only preterm babies or surgical patients following surgery	1
only sub 27 weeks and clinical concerns	1
only those ng fed	1
those below certain gestation/weight and if not on full feeds.	1
volume not routinely measured, unless keeping strict in/out record	1
	90

Supplementary Table 2: Detailed summary of UK NU enteral feeding written guidelines

	Neonatal unit level*	Default feeding method	GRV checking	Threshold for stopping feeds
**1.**	LNU	Bolus feeds with advancement strategy as per SIFT trial	Not specifically mentioned	Aspirate >50% feed volume or green aspirates
**2.**	NICU	Bolus feeds	Routinely measured but no mention of frequency or technique	Aspirate >50% feed volume in previous 6 hours or bilious aspirates
**3.**	NICU	Bolus feeds	Measured but no mention of frequency or how	Aspirates >50% or >1ml<br>If aspirate contains blood or bile.<br>Discard GRV, stop feeds, wait 2 hours and re-assess
**4.**	NICU	Bolus	No mention of frequency or technique	Consider stopping if<br>● pre-feed aspirate >4mls/kg,<br>● heavy bile stained aspirates or<br>● 2 vomits after consecutive feeds
**5.**	LNU	Bolus with advancement as per SIFT trial	GRV aspirated 4 hourly	GRV >25% feeds in previous 4 hours combined with abdominal distention and/or vomiting
**6.**	NICU	No mention	No mention	No mention
**7.**	NICU	Bolus feeds and advanced as per SIFT trial	Check GRV no more than 6 hourly unless concerns	Withhold feeds for 6-12 hours if GRV >40% of feed given or 2 or 3ml (dependant on infant weight), heavily bile or blood stained or abdominal distention
**8.**	LNU	Not stated	Not stated	4 hourly NG aspirates are <25% of total infused in the preceding 4 hours<br>No significant abdominal distension<br>No significant vomiting<br>No bile -<br>stained aspirates
**9.**	NICU	Bolus feeds	Not stated	GRV >50% volume of feeds over last 6 hours or vomit of this size
**10.**	NICU	2 hourly bolus advanced as per SIFT	4-6 hourly	*Action with gastric residuals:*<br>If aspirates 25-50% of total, replace the volume, omit the feed and do not increment.<br>If aspirates >50% of total, stop feeds and medical review.<br>If dark bilious rather than lightly bile stained, stop feeds and medical review
**11.**	NICU	Bolus feeds, 2 risk levels, advanced as per SIFT	Routine measurement of full gastric residuals should be avoided. This should only be done, with discussion, as a part of<br>a full medical/ ANNP	Signs of feed intolerance may include clinical observations such as desaturation and bradycardia events and increased work of breathing, vomiting, abdominal distention and discolouration.
**12.**	SCU	Bolus feeds	Not specified	GRV > 2 hourly amount, vomiting or abdominal distention
**13.**	NICU	Bolus feeds	Not specified	**Medical babies:**<br>2ml/kg of milky gastric residual is not important and should simply be replaced. Where the<br>gastric residual at higher volumes is equivalent to 100% of the bolus, then the feeds should be<br>stopped and a clinical review<br>**Surgical babies:**

				• aspirate <½ feed volume since last aspirate replace the aspirate itself and continue feeding<br>• aspirate ≥ ½ feed volume but <whole feed volume replace half of the aspirate and discard the rest<br>• aspirate ≥whole feed volume since previous aspirate do not replace the aspirate, stop feeding & obtain senior medical and surgical review
**14.**	NICU	Bolus feeds	4 - 6 hourly	Examine and assess the baby if<br>• vomiting,<br>• GRV s >25% of the previous 4 hours total feed volume<br>• residuals are persisting or increasing<br>Small milky / yellow aspirates up to 2-3 mls are frequently normal. They can be replaced, and feeds continued
**15.**	LNU	Bolus feeds	Not specified	Aspirates up to 2-3ml or 50% of the previous 4 hours feed can be normal if the baby is well<br>Aspirates greater than 50% of the previous 4 hours feed or 2-3ml (whichever is greater) discard aspirate, hold feed and try again in 2 hours<br>If aspirate contains blood or bile then stop feeds
**16.**	NICU	Bolus feeds	4 - 6 h	When babies are on any enteral feeds, only aspirate the stomach contents via<br>a gastric tube every 4 - 6 hours, in order to check the residual volume. The assessment of the baby should include any abdominal distension, dark green (bilious) aspirates and bowel opening,<br><br>If <50% of the previous 4 - 6 hour total feed volume is aspirated, then replace the aspirate and continue enteral feeding, provided the baby is<br>otherwise clinically stable<br><br>If >50% of the previous 4 - 6 hour total feed volume is aspirated, then discuss with medical staff; often reasonable to replace the aspirate and omit the feed. If necessary, stop the feeds for 4 - 6 hours; a senior member of the medical / nursing team should then review
**17.**	NICU	Bolus feeds	Q 6 until infant is fully fed	Signs of intolerance<br>1. Vomiting<br>2. Gastric residuals >25% of previous 6<br>hours feed volume, persistent or increasing<br>3. Abdominal distension/increasing abdominal girth<br>4. Increase in stool frequency
**18.**	LNU	Bolus feeds	Not specified	If the aspirates are non-bilious and less than half the volume of previous feed they can be replaced and feeding continued while observing the infant closely<br><br>If the aspirates are bilious or >50% of the previous feed volume, consider withholding the feeds on that occasion and assess for any signs of NEC
**19.**	NICU	Bolus feeds	Not specified	Large volume aspirates or dark green bile stained aspirates, particularly in association with abdominal distension and/or tenderness are a cause for concern. Small milky / yellow aspirates up to 2-3 mls are frequently normal. They can be replaced, and feeds continued
**20.**	LNU	Bolus feeds	Not specified	No mention
**21.**	NICU	Bolus feeds	No more than 4 - 6h	If vomit or GRV exceed 33% of the last feed volume or are more than 3.5 mls in a single aspirate then examine baby<br>Small residuals normal

**22.**	NICU	Bolus feeds	Not specified	Isolated large GRV in the absence of other clinical signs & symptoms should not prevent continued feeding<br>Signs of intolerance:<br>● Vomiting<br>● GRV >30% of previous 5 hours feed<br>● Abdo distention<br>● Unwell baby
**23.**	LNU	Bolus	4-6 hourly	If GRV 25-50% of total, replace the hourly amount, omit the feed and do not increase<br>If GRV >50% of total, stop feeds and medical review
**A.**	Network	Bolus	Not specified	GRV >25% (some >50%) in previous 4 hours in combination with vomiting and abdominal distention + bilious aspirates
**B.**	Network	Bolus feeds	4 hourly	Stop feeds if GRV heavily blood or bile stained<br>No mention of volume
**C.**	Network	Bolus feeds	Not specified	GRV should not be used in isolation to determine feed tolerance<br>Intolerance:<br>Vomiting + GRV >50% in the last 4 hours (especially if increasing) + abdominal distention
**D.**	Network	Bolus feeds advanced as per SIFT	Not specified	Infants 'feed tolerance' assessed with each set of cares (high risk), assess twice daily (mod risk) and before making changes in feed volumes (standard risk)<br>Assessing tolerance:<br>Undigested gastric residuals using a colour chart<br>GRV not used in isolation<br>But vomiting, GRV >25% of feed volume in last 4 hours + bloody or bilious residuals + abdominal distention
**E**	Network	Bolus	Assess GRV 4 - 6 hourly depending on cares	If GRV >50% of total, stop feeds and medical review<br>If GRV 25-50% of total, replace the hourly amount, omit the feed and do not increase<br>An appropriate GRV is <25% of preceding volume since last replacement of GRV<br>Replace GRV in full<br>A GRV >25% but <1.5mls unlikely to be problematic<br>A GRV of 25-50% is high, but acceptable if well, replace only normal hourly volume and continue feeds but do not increase<br>A GRV >50% is excessive, perform clinical exam, if acceptable hourly volume can be replaced but feed withheld

*Neonatal Unit Level determined by NNAP 2017 report (https://www.hqip.org.uk/resource/national-neonatal-audit-programme-2017-annual-report-on-2016-data/)

Abbreviations: GRV = Gastric Residual Volume; SIFT = Speed of Increasing of milk Feeds Trial(23), NICU = Neonatal Intensive Care Unit, LNU = Local Neonatal Unit, SCU = Special Care Unit.
