# Supplementary material for: Gastric residual volume measurement in British neonatal intensive care units: a survey of practice
Source: BMJ Paediatr Open. 2020 Aug 7;4(1):e000601. doi: 10.1136/bmjpo-2019-000601 (PMC7418677; doi:10.1136/bmjpo-2019-000601)
Supplement: Author's manuscript [file bmjpo-2019-000601.draft_revisions.pdf]

BMJ Paediatrics Open

BMJ Paediatrics Open is committed to open peer review. As part of this commitment we make the peer review history of every article we publish publicly available.

When an article is published we post the peer reviewers' comments and the authors' responses online. We also post the versions of the paper that were used during peer review. These are the versions that the peer review comments apply to.

The versions of the paper that follow are the versions that were submitted during the peer review process. They are not the versions of record or the final published versions. They should not be cited or distributed as the published version of this manuscript.

BMJ Paediatrics Open is an open access journal and the full, final, typeset and author-corrected version of record of the manuscript is available on our site with no access controls, subscription charges or pay-per-view fees (http://bmjpaedsopen.bmj.com).

If you have any questions on BMJ Paediatrics Open's open peer review process please email info.bmjpo@bmj.com

BMJ Paediatrics Open

Gastric Residual Volume measurement in UK neonatal intensive care units: a survey of practice

Journal:	*BMJ Paediatrics Open*
Manuscript ID	bmjpo-2019-000601
Article Type:	Original research
Date Submitted by the Author:	19-Feb-2020
Complete List of Authors:	Dorling, Jon; Dalhousie University - Faculty of Medicine, Division of Neonatal-Perinatal Medicine<br>Tume, Lyvonne; University of Salford, Child Health<br>Arch, Barbara; University of Liverpool, Liverpool Clinical Trials Unit<br>Woolfall, Kerry; University of Liverpool, Health Services Research<br>Latten, Lynne; Alder Hey Children's Hospital<br>Roper, Louise; University of Liverpool, Department of Health Services Research<br>Deja, Elizabeth; University of Liverpool, Health Services Research<br>Pathan, Nazima; University of Cambridge, Paediatrics<br>Eccleson, Helen; University of Liverpool, Liverpool Clinical Trials Unit<br>Hickey, Helen; University of Liverpool, Liverpool Clinical Trials Unit<br>Brown, Michaela; University of Liverpool, Liverpool Clinical Trials Unit<br>Beissel, Anne; Hôpital Femme Mère Enfant, Neonatal Intensive Care Unit<br>Andrzejewska, izabela ; Chelsea and Westminster Healthcare NHS Trust, Neonatal Unit<br>Valla, Frederic; Hospices Civils de Lyon, Université Claude-Bernard Lyon 1, Service de réanimation pédiatrique, Hôpital Femme-Mère-Enfant<br>Gale, Chris; Imperial College London, Academic Neonatal Medicine
Keywords:	Measurement, Neonatology, Gastroenterology

SCHOLARONE™
Manuscripts

I, the Submitting Author has the right to grant and does grant on behalf of all authors of the Work (as defined in the below author licence), an exclusive licence and/or a non-exclusive licence for contributions from authors who are: i) UK Crown employees; ii) where BMJ has agreed a CC-BY licence shall apply, and/or iii) in accordance with the terms applicable for US Federal Government officers or employees acting as part of their official duties; on a worldwide, perpetual, irrevocable, royalty-free basis to BMJ Publishing Group Ltd ("BMJ") its licensees and where the relevant Journal is co-owned by BMJ to the co-owners of the Journal, to publish the Work in this journal and any other BMJ products and to exploit all rights, as set out in our licence.

The Submitting Author accepts and understands that any supply made under these terms is made by BMJ to the Submitting Author unless you are acting as an employee on behalf of your employer or a postgraduate student of an affiliated institution which is paying any applicable article publishing charge ("APC") for Open Access articles. Where the Submitting Author wishes to make the Work available on an Open Access basis (and intends to pay the relevant APC), the terms of reuse of such Open Access shall be governed by a Creative Commons licence – details of these licences and which Creative Commons licence will apply to this Work are set out in our licence referred to above.

Other than as permitted in any relevant BMJ Author's Self Archiving Policies, I confirm this Work has not been accepted for publication elsewhere, is not being considered for publication elsewhere and does not duplicate material already published. I confirm all authors consent to publication of this Work and authorise the granting of this licence.

Confidential: For Review Only

TITLE PAGE

Gastric Residual Volume measurement in UK neonatal intensive care units: a survey of practice

Authors: Dorling J, Tume LN, Arch B, Woolfall K, Latten L, Roper L, Deja, E, Pathan N, Eccleson H,

Hickey H, Brown M, Beissel A, Andrzejewska I, Valla FV, Gale C

Corresponding author:

Jon Dorling FRCPCH, MD
Professor of Pediatrics and Division Head
Dalhousie University and IWK Health Centre
Division of Neonatal-Perinatal Medicine
5850/5980 University Avenue
P.O. Box 9700
Halifax, Nova Scotia, Canada, B3K 6R8
Jon.dorling@iwk.nshealth.ca

Lyvonne Tume, RN, PhD
Reader in Child Health,
University of Salford, Manchester UK
Frederick Road Campus, M6 6PU
Email: l.n.tume@salford.ac.uk

Barbara Arch MSc
Statistician
Liverpool Clinical Trials Unit
University of Liverpool
Institute in the Park
Alder Hey Children's NHS Foundation Trust
Liverpool
L12 2AP
barbara.arch@liverpool.ac.uk

Kerry Woolfall BA (Hons), MA, PhD
Senior Lecturer
Department of Health Services Research
University of Liverpool
Block B, Room B112, 1st Floor Waterhouse Building,
Liverpool L69 3GL
k.woolfall@liverpool.ac.uk

Lynne Latten Bsc (Hons) RD
Advanced Paediatric Dietitian Critical Care
Alder Hey Children's Hospital
Liverpool
L12 2AP
Lynne.Latten@alderhey.nhs.uk

Confidential: For Review Only

Louise Roper BSc, MSc, CPsychol, PhD
Chartered Health Psychologist,
Department of Health Services Research,
Block B, 1st Floor, Waterhouse Building,
University of Liverpool
Liverpool L69 3GX
Louise.Roper@liverpool.ac.uk

Elizabeth Deja BSc, MSc, PhD
Research Associate
Department of Health Services Research,
Block B, 1st Floor, Waterhouse Building,
University of Liverpool
Liverpool L69 3GX
bdeja1@liverpool.ac.uk

Nazima Pathan FRCPCH PhD
Consultant and University Lecturer in Paediatric Intensive Care
University of Cambridge
Addenbrooke's Hospital
Cambridge
CB2 0QQ
np409@cam.ac.uk

Helen Eccleson Bsc (Hons)
Trial Coordinator
Liverpool Clinical Trials Unit University of Liverpool
Institute in the Park
Alder Hey Children's NHS Foundation Trust
Liverpool
L12 2AP
Helen.Eccleson@liverpool.ac.uk

Helen Hickey PgD (Dist)
Head of Trial Management
Liverpool Clinical Trials Unit University of Liverpool
Institute in the Park
Alder Hey Children's NHS Foundation Trust
Liverpool
L12 2AP
Email: h.hickey@liverpool.ac.uk

Michaela Brown MSc
Senior Statistician
Liverpool Clinical Trials Unit
University of Liverpool
Institute in the Park
Alder Hey Children's NHS Foundation Trust
Liverpool
L12 2AP
Michaela.Brown@liverpool.ac.uk

Confidential: For Review Only

Anne Beissel MD
Consultant in Neonatal Intensive Care Unit
Neonatal Intensive Care Unit
Hôpital Femme Mère Enfant, Hospices Civils de Lyon
59 bd Pinel, 69500 Lyon-Bron, France
anne.beissel@chu-lyon.fr

Izabela Andrzejewska, RN MSc
Neonatal Unit Coordinator/Neonatal Research Nurse
Chelsea and Westminster Hospital
Neonatal Unit
3rd Floor, lift bank D
369 Fulham Road
London
SW10 9NH
i.andrzejewska@chelwest.nhs.uk

Frédéric V Valla MD MSc
Consultant in Pediatric Intensive Care Medicine
Pediatric Intensive Care Unit
CarMEN INSERM UMR 1060 Equipe INFOLIP
Hôpital Femme Mère Enfant, Hospices Civils de Lyon
59 bd Pinel, 69500 Lyon-Bron, France
Frederic.valla@chu-lyon.fr
and
Visiting Research Fellow
University of the West of England
Faculty of Health & Applied Sciences,
Blackberry Hill,
Bristol, BS16 1DD, UK

Chris Gale MRCPCH PhD
Reader in Neonatal Medicine
Imperial College London, Chelsea and Westminster Hospital campus,
London, SW10 9NH
Christopher.gale@imperial.ac.uk

This work was supported by NIHR HTA programme, grant number 16/94/02

Confidential: For Review Only

Dr. Dorling reports grants from National Institute for Health Research (NIHR), during the conduct of the study; grants from National Institute for Health Research, grants from Nutrinia, outside the submitted work.

Mrs. Latten reports grant from National Institute for Health Research, during the conduct of the study.

Dr. Deja reports grants from NIHR Health Technology Assessment Programme during the conduct of the study.

H. Eccleson reports grants from NIHR Health Technology Assessment during the conduct of the study.

H. Hickey reports this grant from NIHR Health Technology Assessment, during the conduct of the study.

M. Brown reports grants from NIHR HTA during the conduct of the study.

Dr. Valla reports personal fees from Baxter, personal fees from Nutricia, outside the submitted work.

Dr. Tume reports grants from NIHR during the conduct of the study.

Dr. Gale reports grants from National Institute for Health Research (NIHR), during the conduct of the study; grants from National Institute for Health Research, grants from Medical Research Foundation, grants from Mason Medical Research Foundation, grants and personal fees from Chiesi Pharmaceuticals, grants from Rosetrees Foundation, grants from Canadian Institute for Health Research, outside the submitted work

All other authors indicate that they have nothing to disclose.

All authors contributed to the study. JD and LT planned the study and incorporated comments from all members on the study design, conduct, interpretation and reporting of the work. BA analysed quantitative data, KW, ED and LR analysed the open answer responses, LT and JD analysed the guideline content. JD is responsible for the overall content as guarantor.

Confidential: For Review Only

Gastric Residual Volume measurement in UK neonatal intensive care units: a survey of practice

Abstract

Objective: Despite little evidence, the practice of routine gastric residual volume (GRV) measurement to guide enteral feeding in neonatal units is widespread. Due to increased interest in this practice, and to examine trial feasibility, we aimed to determine enteral feeding and GRV measurement practices in United Kingdom (UK) neonatal units.

Design & Setting: An online survey was distributed via email to all neonatal units and networks in England, Scotland and Wales. A clinical nurse, senior doctor and dietitian were invited to collaboratively complete the survey and submit a copy of relevant guidelines.

Results: 95/184 (51.6%) approached units completed the survey, 81/95 (85.3%) reported having feeding guidelines and 28 guidelines were submitted for review. The majority of units used intermittent (90/95) gastric feeds as their primary feeding method. 42/95 units reported specific guidance for measuring and interpreting GRV. 20/90 units measured GRV before every feed, 39/90 at regular time-intervals (most commonly 4-6 hourly 35/39) and 26/90 when felt to be clinically indicated. Most units reported uncertainty on the utility of aspirate volume for guiding feeding decisions; 13/90 reported that aspirate volume affected decisions 'very much'. In contrast, aspirate colour was reported to affect decisions 'very much' by 37/90 of responding units. Almost half, 44/90, routinely returned aspirates to the stomach.

Conclusions: Routine GRV measurement is part of standard practice in UK neonatal units, although there was inconsistency in how frequently to measure or how to interpret the aspirate. Volume was considered less important than colour of the aspirate.

Keywords: newborn; infant; feeding; nutrition; feasibility study, questionnaire; critically ill

Confidential: For Review Only

INTRODUCTION

The gastric residual volume (GRV) is the volume of the entire stomach contents, obtained by aspiration with a syringe in order to assess feeding tolerance. It provides information on the volume and colour of fluid, and is distinct from the aspiration of a small amount of fluid for pH testing to confirm feeding tube position(1). There is a paucity of evidence to support routine measurement of GRV to direct and guide enteral feeding, and the practice is increasingly being questioned in neonatal units (1–5). For many clinicians, however, this parameter is a fundamental part of the definition and diagnosis of feed intolerance.(6) The rationale for routinely measuring GRV in the neonatal setting is for the early identification of necrotizing enterocolitis (NEC) and prevention of complications such as vomiting or aspiration, by withholding or reducing feed volumes. (1,7,8) Routine measurement, could, however, cause harm, for example through direct injury of the gastric mucosa, discarding gastric juices, medications and hormones, and by delaying enteral feeding and prolonging parenteral nutrition.(4,9,10) Furthermore, measurement of GRV has been shown to be inaccurate and affected by the position of the baby and the tube, hence it is not a useful surrogate marker for delayed gastric emptying in premature infants (11–14).

In this study, we aimed to identify current practice around GRV measurement in the United Kingdom (UK). In addition, we sought to delineate enteral feeding practices in UK neonatal units in relation to GRV, and to identify a 'control arm' for a future trial comparing no routine GRV measurement (the intervention) to routine GRV measurement.

METHODS

A survey instrument was developed by the research team to explore current practices around GRV measurement and general enteral feeding practices in neonatal units. The intention was to use these survey findings alongside a review of neonatal unit guidelines to establish current practice. A 10 item

Confidential: For Review Only

closed question survey (tick-box responses) with optional free text response, and nine open-ended questions was developed by the researchers. The survey was piloted for face validity with 10 staff (doctors, dietitians, nurses). Minor wording adjustments were made to improve clarity, before the 19-item survey (Supplementary material) was entered onto the survey platform and retested by the study team.

The survey focussed on three domains: general enteral feeding and nutrition practices in the respondents' unit, the GRV measurement technique used in the respondents' unit, and clinical management in response to GRV. The survey invitation requested that a senior doctor, a clinical nurse and a dietitian complete the survey collaboratively and submit one response per unit, and requested that any relevant written guidelines or protocols be submitted. Unit name was collected, to target non-responders and check for duplicates; three reminders were sent to maximise response rates. Our target response rate was 70%.

All National Health Service (NHS) neonatal units in England, Scotland and Wales were approached during May and June 2018 using email invitations directed at 184 neonatal teams (some neonatal teams cover multiple neonatal units) sent through a national research collaboration, United Kingdom Neonatal Collaborative (UKNC), and a multi-disciplinary professional network, The Neonatal Nutrition Network (N3). Units in Northern Ireland were not contacted as they are not part of the UKNC. Study data were collected and managed using REDCap electronic data capture tools hosted at the University of Liverpool.(15) Data were summarised using descriptive statistics for quantitative data and a mix of thematic and content analysis for qualitative free text data.(16,17) Following this, the neonatal unit guidelines were reviewed and summarized. Ethical approval for the study was provided by the University of the West of England (Reference: HAS.18.04.144).
There was no direct patient or public involvement in the work presented in this manuscript as it involved surveying clinicians on their clinical practice. Other aspects of the research not reported

Confidential: For Review Only

here had substantial input as they involved qualitative interviews and consensus gathering (NIHR HTA journals in press).

RESULTS

95 of 184 (51.6%) neonatal units in the UK excluding Northern Ireland completed the survey. These consisted of 40 Neonatal Intensive Care Units (NICUs), 42 Local Neonatal Units (LNUs) and 13 Special Care Baby Units (SCBUs) giving response rates of 71.4%, 47.2% and 33.3% of the NICUs, LNUs and SCBUs respectively. Seventeen of a possible eighteen NICUs caring for both surgical and medical patients responded, as did 23 NICUs caring for medical cases only. LNUs and SCBUs do not provide early post-operative care in the UK.

Survey responses were received from senior doctors (81/95, 85.3%); nurses (51/95, 53.7%) and dietitians (9/95, 9.5%). Most (81/95, 85.3%) responding units reported written enteral feeding guidance and 28 unit or local neonatal network guidelines were sent to the author (Supplementary table). Enteral feeding was typically delivered intermittently (90/95, 94.7%) rather than continuously (5/95, 5.3%). 42/95 units (44.2%) reported having written guidance for measurement and interpretation of gastric residual volumes. Ninety units answered questions about the management of non-surgical babies. When asked about how often GRV is measured, 20/90 units (22.2%) measured aspirates before every feed, 26/90 (28.9%) when it was felt to be clinically indicated, and 39/90 (43.3%) measured GRV at regular time intervals (most commonly 4-6 hourly 35/39 (89.7%), but all more frequent than once per day). One unit had no guidelines on this, and 4/90 (4.4%) reported that they did not measure GRV. Among units that reported having written GRV measurement guidance, 13/39 (43.3%) indicated that the guidance was 'always', and 17/39 (38.6%) 'usually' followed, however free text responses suggested that practice was *"very variable depending on the nurse looking after the baby"* (Unit 3, surgical and medical unit). The bedside nurse

Confidential: For Review Only

most commonly made decisions in relation to GRV results, 56/90 (62.2%), followed by middle grade doctors, 41/90 (45.6%), and the senior nurse in charge of shift, 26/90 (28.9%).

Responding units had mixed views on how useful the volume of the aspirate was for guiding feeding decisions (Figure): just 13/90 (14.4%) of units reporting that volume affected clinical decision-making 'very much' and the most frequent response was an intermediate score. The colour of the aspirate was felt to be more important: 37/90 (41.1%) of units reporting that colour influenced clinical decisions 'very much' and this was the most frequent response. More detail was obtained from 74 open text responses to this question. A large volume of aspirate was commonly described as a concern, which would often lead to a clinical review of a baby's condition and subsequent consideration of the how much milk the baby is receiving. The threshold for prompting a feeding review was reported to vary. Some units stated that aspirates over 50% of the feed would "prompt a review" (Unit 8, NICU surgical and medical), whilst others stated ">25% of feed given in previous 6 hours" (Unit 18, NICU medical only), if exceeds "25% of the previous 4 hours' feed volume" (Unit 22, NICU medical only) or "If >25% of the feed volume given since the last assessment was made" (Unit 25, NICU medical only).

Almost half, 44/90 (48.9%), routinely returned aspirates to the stomach. 72 nurses gave reasons for seeking medical advice: 55/72 (76.4%) cited increased or large volume GRVs, 52/72 (72.2%) cited bilious colour of the residual, or a change in colour. Other reasons were blood stained aspirates 16/72 (22.2%), concerns about condition of baby, such as desaturations 16/72 (22.2%), abdominal distention 11/72 (15.3%), and vomiting 5/72 (6.9%). In free text responses, units stated that that a dark or bilious colour would *"trigger medical review [by a] Middle Grade or Consultant"* (Unit 22, NICU medical only), whilst some described how feeds would be stopped: *"Green aspirate - assess baby and feeds withheld"* (Unit 60, LNU).

Confidential: For Review Only

Guideline analysis (Table 2 & Supplementary Table) revealed that 19 of 28 (67.8%) guidelines specified a volume of aspirate at which to consider stopping feeds using a defined proportion of the previous feed. Six guidelines specified this threshold as 25% or more of the previous feed, eight guidelines specified 50% or more, while five guidelines used a level between these. Fourteen guidelines mentioned the bilious green colouring of GRV being an indication to stop enteral feeds, while five mentioned blood staining as being important. Vomiting and abdominal distension were also considered important for guiding management being mentioned by 13 and 12 guidelines respectively.

DISCUSSION

The results of this survey confirm mixed practice in neonatal units across the United Kingdom for both monitoring GRV and in how findings are used to make decisions about enteral feeding. This survey also identifies that around half of UK neonatal units use GRV as a parameter to guide enteral feeding advancement. Health professionals' views around the importance of the volume compared to the colour of the GRV were inconsistent and importance was defined at different thresholds. Aspirate colour was cited more often as important than volume of gastric residuals, however the importance of aspirate colour was inconsistent; some unit guidelines specified actions based on bilious or blood staining of the secretions whereas others did not mention them, and many unit guidelines referred to not returning aspirates that were bilious (green) or bloody (red) in colour. Change in aspirate colour was viewed as a potential indicator of NEC in preterm neonates in this survey, but this and many aspects of residual evaluation are unsubstantiated by high quality evidence.(5)

The mixed views elicited on interpreting volume are consistent with the paucity of evidence for routine GRV measurement, and support randomised trials to assess whether aspirating the stomach contents is a useful practice(4,7,9). Although it might be beneficial to stop measuring

Confidential: For Review Only

GRV in neonatal units, some health professionals believe their measurement can help to identify NEC earlier despite the absence of evidence to support this presumption. Recent results from small studies involving preterm infants suggests that not measuring GRV is not associated with an increase in the risk of NEC and might reduce the time to achieve full enteral feeds (3,4,9,18), however these studies were underpowered to detect even large relative differences in rare outcomes like NEC. Adequate power to definitively assess NEC would require a trial of thousands of participants rather than the 230 randomised participants studied to date(2,3). Routine monitoring of GRV does however add to nursing workload and may lead to other direct harms to the infant. Given the widespread use of this practice, a future trial would need to demonstrate the safety of both monitoring and not monitoring GRV.

The routine measurement of GRV is based on the presumption that GRV are an accurate representation of the residual gastric contents. Laboratory-based simulation studies undermine this presumption, however, by demonstrating that GRV inaccurately measure gastric contents.(19,20) The GRV obtained is widely influenced by a number of factors such as the syringe size, gastric tube size and material, aspiration pressure, viscosity of aspirate, and both the position of the tube tip in the stomach and of the neonate.(21) Furthermore, when decision-making is based on volume, clinicians fail to consider the impact of gastric secretions produced during the digestion process.(22)

This study has limitations: firstly, as with any survey, responses may not reflect actual practice. However, we were able to obtain a summary of what ought to happen by reviewing unit guidelines. Secondly, it is a weakness of the study that there were low responses from the smaller neonatal units. The results might therefore over-represent the views of larger NICU units.

Confidential: For Review Only

CONCLUSIONS

The routine and frequent measurement of GRV is embedded in enteral feeding practice and guidelines in UK neonatal units, despite a lack of evidence and questionable accuracy of this parameter. For many units, GRV is integral to the assessment of feed tolerance/intolerance with bilious colouring of the aspirate and presence of blood being considered important. This study has identified current practice around GRV measurement in UK neonatal units, and supports examination of the benefits and harms of GRV in an adequately powered, randomised, controlled trial.

Acknowledgements: We thank all the neonatal units who took part in this survey.

Confidential: For Review Only

“What is already known on this topic” – followed by a maximum of 3 brief statements (no more than 25 words per statement);

1. The gastric residual volume is the volume of the entire stomach contents, obtained by aspiration with a syringe in order to assess feeding tolerance.

2. It is unclear if the routine measurement of gastric residual volume is beneficial or harmful in preterm infants.

3. The rationale for routinely measuring GRV is an attempt at identifying early of necrotizing enterocolitis and prevention of complications by withholding or reducing feed volumes.

“What this study adds” – followed by a maximum of 3 brief statements (no more than 25 words per statement).

1. This study demonstrates mixed practice for residual measurements across neonatal units in the UK.
2. Aspirate colour was reported as affect decisions mpore often ion comparsion to residual volume.
3. A randomised trial appears feasible in the United Kingdom given the variation in practice and willingness of respondents to randomise to measuring or not measuring.

Confidential: For Review Only

REFERENCES

1. Mihatsch WA, von Schoenaich P, Fahnenstich H, Dehne N, Ebbecke H, Plath C, et al. The Significance of Gastric Residuals in the Early Enteral Feeding Advancement of Extremely Low Birth Weight Infants. Pediatrics. 2002 Mar;109(3):457–459.

2. Singh B, Rochow N, Chessell L, Wilson J, Cunningham K, Fusch C, et al. Gastric Residual Volume in Feeding Advancement in Preterm Infants (GRIP Study): A Randomized Trial. J Pediatr. 2018;200:79-83.e1.

3. Parker LA, Weaver M, Torrazza RJM, Shuster J, Li N, Krueger C, et al. Effect of Gastric Residual Evaluation on Enteral Intake in Extremely Preterm Infants: A Randomized Clinical Trial. JAMA Pediatr. 2019 Jun 1;173(6):534–43.

4. Riskin A, Cohen K, Kugelman A, Toropine A, Said W, Bader D. The Impact of Routine Evaluation of Gastric Residual Volumes on the Time to Achieve Full Enteral Feeding in Preterm Infants. J Pediatr. 2017;189:128–34.

5. Li Y-F, Lin H-C, Torrazza RM, Parker L, Talaga E, Neu J. Gastric Residual Evaluation in Preterm Neonates: A Useful Monitoring Technique or a Hindrance? Pediatr Neonatol. 2014 Oct;55(5):335–40.

6. Moore TA, Wilson ME. Feeding Intolerance: A Concept Analysis. Adv Neonatal Care. 2011 Jun;11(3):149.

7. Cobb BA, Carlo WA, Ambalavanan N. Gastric Residuals and Their Relationship to Necrotizing Enterocolitis in Very Low Birth Weight Infants. Pediatrics. 2004 Jan 1;113(1):50–3.

8. Bertino E, Giuliani F, Prandi G, Coscia A, Martano C, Fabris C. Necrotizing enterocolitis: risk factor analysis and role of gastric residuals in very low birth weight infants. J Pediatr Gastroenterol Nutr. 2009 Apr;48(4):437–42.

9. Torrazza RM, Parker LA, Li Y, Talaga E, Shuster J, Neu J. The value of routine evaluation of gastric residuals in very low birth weight infants. J Perinatol Off J Calif Perinat Assoc. 2015 Jan;35(1):57–60.

10. Tume LN, Bickerdike A, Latten L, Davies S, Lefèvre MH, Nicolas GW, et al. Routine gastric residual volume measurement and energy target achievement in the PICU: a comparison study. Eur J Pediatr. 2017 Dec;176(12):1637–44.

11. Geraldo V, Pyati S, Joseph T, Pildes RS. Gastric Residual (GR): Reliability of the Measurement. 888. Pediatr Res. 1997 Apr 1;41(S4):150.

12. Parker L, Torrazza RM, Li Y, Talaga E, Shuster J, Neu J. Aspiration and evaluation of gastric residuals in the neonatal intensive care unit: state of the science. J Perinat Neonatal Nurs. 2015 Mar;29(1):51–9; quiz E2.

13. Shulman RJ, Ou C-N, Smith EO. Evaluation of potential factors predicting attainment of full gavage feedings in preterm infants. Neonatology. 2011;99(1):38–44.

Confidential: For Review Only

14. Jadcherla SR, Kliegman RM. Studies of feeding intolerance in very low birth weight infants: definition and significance. Pediatrics. 2002 Mar;109(3):516–7.

15. Harris PA, Taylor R, Thielke R, Payne J, Gonzalez N, Conde JG. Research electronic data capture (REDCap)--a metadata-driven methodology and workflow process for providing translational research informatics support. J Biomed Inform. 2009 Apr;42(2):377–81.

16. Hsieh H-F, Shannon SE. Three approaches to qualitative content analysis. Qual Health Res. 2005 Nov;15(9):1277–88.

17. Braun V, Clarke V. Using thematic analysis in psychology. Qual Res Psychol. 2006 Jan 1;3(2):77–101.

18. al BS et. Gastric Residual Volume in Feeding Advancement in Preterm Infants (GRIP Study): A Randomized Trial | Kopernio [Internet]. [cited 2019 Apr 25]. Available from: https://kopernio.com/viewer?doi=10.1016/j.jpeds.2018.04.072&token=WzExNzcyOCwiMTAuMTAxNi9qLmpwZWRzLjIwMTguMDQuMDcyIl0.58y70TAsh-b7Ky3TokWFqsMawr0

19. Bartlett Ellis RJ, Fuehne J. Examination of accuracy in the assessment of gastric residual volume: a simulated, controlled study. JPEN J Parenter Enteral Nutr. 2015 May;39(4):434–40.

20. Lin HC, Van Citters GW. Stopping enteral feeding for arbitrary gastric residual volume may not be physiologically sound: results of a computer simulation model. JPEN J Parenter Enteral Nutr. 1997 Oct;21(5):286–9.

21. Sangers H, Jong PM de, Mulder SE, Stigter GD, Berg CM van den, Pas AB te, et al. Outcomes of gastric residuals whilst feeding preterm infants in various body positions. J Neonatal Nurs. 2013 Dec 1;19(6):337–41.

22. Tume LN, Latten L, Kenworthy L. Paediatric intensive care nurses' decision-making around gastric residual volume measurement. Nurs Crit Care. 2017 Sep;22(5):293–7.

23. Abbott J, Berrington J, Bowler U, Boyle E, Dorling J, Embleton N, et al. The Speed of Increasing milk Feeds: a randomised controlled trial. BMC Pediatr. 2017 Jan 28;17:39.

Figure and Table legends

Figure: Perceived Importance of Aspirate Volume and Colour for making feeding decisions

Table 1: Survey results – General feeding practices for all babies

Table 2: Survey results - GRV practices specific to the management of medical babies

Supplementary Table: Detailed summary of UK NU enteral feeding written guidelines

Confidential: For Review Only

Table 1: Survey results – General feeding practices for all babies (n=95)

Practice	N (%)
Units had written feeding guidelines/protocol	81 (85.3%)
Standard NG feeds were intermittent bolus (not continuous)	90 (94.7%)
There was specific guidance about how Gastric Residual Volume should be measured and interpreted - for example a protocol or guideline	42 (44.2%)
NICUs that care for surgical and medical babies (n=17):	
Gastric Residual Volume measurement differs between the medical and surgical babies	5/17 (29.4%)

Confidential: For Review Only

Table 2: Survey results - GRV practices specific to the management of medical babies (n=90)

Survey question	N (%)
**How often do staff in your unit measure GRV?**	
Once a day	0 (0%)
Before every feed	20 (22.2%)
Only when clinically indicated	26 (28.9%)
At regular intervals	39 (43.4%)
At least every 3,4 or 6 hrs	*35/39*
GRV is not measured	4 (4.4%)
**Is the specific guidance for GRV measurement followed and actually undertaken as per protocol – Only asked of units with specific guidance for GRV measurement (n=39)?**	
Always	13 (43.3%)
Usually	17 (38.6%)
Often	4 (10.3%)
Rarely / Never	5 (12.8%)
**Who usually decides what to do with concerning GRV aspirates in the first instance? (more than one response allowed)**	
Senior Doctor (Consultant)	13 (14.4%)
Middle Grade Doctor (SpR)	41 (45.6%)
Junior Grade Doctor (SHO)	18 (20.0%)
Bedside Nurse	56 (62.2%)
Nurse in charge of shift (senior nurse)	26 (28.9%)
**How much does volume of the aspirate affect your decision around GRV?**	
1 Not at all	5 (5.6%)
2	11 (12.2%)
3	40 (44.4%)
4	21 (23.3%)
5 (Very much)	13 (14.4%)
**How much does colour of the aspirate affect your decision around GRV?**	
1 Not at all	3 (3.3%)
2	6 (6.7%)
3	16 (17.8%)
4	28 (31.1%)
5 (Very much)	37 (41.1%)
**What do you do with obtained GRV: return or discard?**	
Return	44 (48.9%)
Discard	7 (7.8%)
Other	39 (43.3%)

Abbreviations: GRV Gastric Residual Volume

Confidential: For Review Only

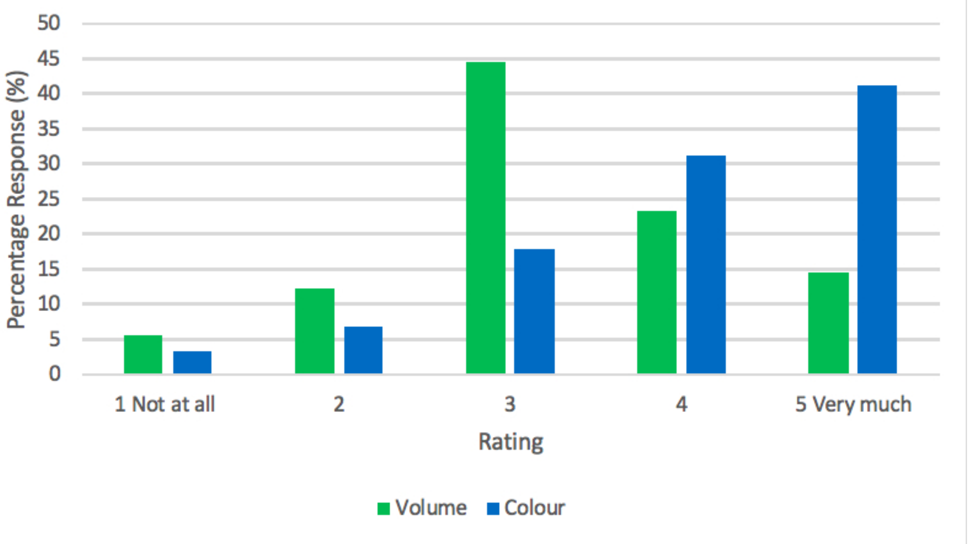
50
45
40
35
30
25
20
15
10
5
0
Percentage Response (%)
1 Not at all
2
3
4
5 Very much
Rating
Volume
Colour

Supplementary Table: Detailed summary of UK NU enteral feeding written guidelines

	Neonatal unit level*	Default feeding method	GRV checking	Threshold for stopping feeds
**1.**	LNU	Bolus feeds with advancement strategy as per SIFT trial	Not specifically mentioned	Aspirate >50% feed volume or green aspirates
**2.**	NICU	Bolus feeds	Routinely measured but no mention of frequency or technique	Aspirate >50% feed volume in previous 6 hours or bilious aspirates
**3.**	NICU	Bolus feeds	Measured but no mention of frequency or how	Aspirates >50% or >1ml<br>If aspirate contains blood or bile.<br>Discard GRV, stop feeds, wait 2 hours and re-assess
**4.**	NICU	Bolus	No mention of frequency or technique	Consider stopping if<br>• pre-feed aspirate >4mls/kg,<br>• heavy bile stained aspirates or<br>• 2 vomits after consecutive feeds
**5.**	LNU	Bolus with advancement as per SIFT trial	GRV aspirated 4 hourly	GRV >25% feeds in previous 4 hours combined with abdominal distention and/or vomiting
**6.**	NICU	No mention	No mention	No mention
**7.**	NICU	Bolus feeds and advanced as per SIFT trial	Check GRV no more than 6 hourly unless concerns	Withhold feeds for 6-12 hours if GRV >40% of feed given or 2 or 3ml (dependant on infant weight), heavily bile or blood stained or abdominal distention
**8.**	LNU	Not stated	Not stated	4 hourly NG aspirates are <25% of total infused in the preceding 4 hours<br>No significant abdominal distension<br>No significant vomiting<br>No bile -<br>stained aspirates
**9.**	NICU	Bolus feeds	Not stated	GRV >50% volume of feeds over last 6 hours or vomit of this size
**10.**	NICU	2 hourly bolus advanced as per SIFT	4-6 hourly	*Action with gastric residuals:*<br>If aspirates 25-50% of total, replace the volume, omit the feed and do not increment.<br>If aspirates >50% of total, stop feeds and medical review.<br>If dark bilious rather than lightly bile stained, stop feeds and medical review
**11.**	NICU	Bolus feeds, 2 risk levels, advanced as per SIFT	Routine measurement of full gastric residuals should be avoided. This should only be done, with discussion, as a part of<br>a full medical/ ANNP	Signs of feed intolerance may include clinical observations such as desaturation and bradycardia events and increased work of breathing, vomiting, abdominal distention and discolouration.
**12.**	SCU	Bolus feeds	Not specified	GRV > 2 hourly amount, vomiting or abdominal distention
**13.**	NICU	Bolus feeds	Not specified	**Medical babies:**<br>2ml/kg of milky gastric residual is not important and should simply be replaced. Where the<br>gastric residual at higher volumes is equivalent to 100% of the bolus, then the feeds should be<br>stopped and a clinical review<br>**Surgical babies:**<br>• aspirate <½ feed volume since last aspirate replace the aspirate itself and continue feeding

				• aspirate ≥ ½ feed volume but <whole feed volume replace half of the aspirate and discard the rest<br>• aspirate ≥whole feed volume since previous aspirate do not replace the aspirate, stop feeding & obtain senior medical and surgical review
**14.**	NICU	Bolus feeds	4 - 6 hourly	Examine and assess the baby if<br>• vomiting,<br>• GRV s >25% of the previous 4 hours total feed volume<br>• residuals are persisting or increasing<br>Small milky / yellow aspirates up to 2-3 mls are frequently normal. They can be replaced, and feeds continued
**15.**	LNU	Bolus feeds	Not specified	Aspirates up to 2-3ml or 50% of the previous 4 hours feed can be normal if the baby is well<br>Aspirates greater than 50% of the previous 4 hours feed or 2-3ml (whichever is greater) discard aspirate, hold feed and try again in 2 hours<br>If aspirate contains blood or bile then stop feeds
**16.**	NICU	Bolus feeds	4 - 6 h	When babies are on any enteral feeds, only aspirate the stomach contents via<br>a gastric tube every 4 - 6 hours, in order to check the residual volume. The assessment of the baby should include any abdominal distension, dark green (bilious) aspirates and bowel opening,<br><br>If <50% of the previous 4 - 6 hour total feed volume is aspirated, then replace the aspirate and continue enteral feeding, provided the baby is<br>otherwise clinically stable<br><br>If >50% of the previous 4 - 6 hour total feed volume is aspirated, then discuss with medical staff; often reasonable to replace the aspirate and omit the feed. If necessary, stop the feeds for 4 - 6 hours; a senior member of the medical / nursing team should then review
**17.**	NICU	Bolus feeds	Q 6 until infant is fully fed	Signs of intolerance<br>1. Vomiting<br>2. Gastric residuals >25% of previous 6<br>hours feed volume, persistent or increasing<br>3. Abdominal distension/increasing abdominal girth<br>4. Increase in stool frequency
**18.**	LNU	Bolus feeds	Not specified	If the aspirates are non-bilious and less than half the volume of previous feed they can be replaced and feeding continued while observing the infant closely<br><br>If the aspirates are bilious or >50% of the previous feed volume, consider withholding the feeds on that occasion and assess for any signs of NEC
**19.**	NICU	Bolus feeds	Not specified	Large volume aspirates or dark green bile stained aspirates, particularly in association with abdominal distension and/or tenderness are a cause for concern. Small milky / yellow aspirates up to 2-3 mls are frequently normal. They can be replaced, and feeds continued
**20.**	LNU	Bolus feeds	Not specified	No mention
**21.**	NICU	Bolus feeds	No more than 4 - 6h	If vomit or GRV exceed 33% of the last feed volume or are more than 3.5 mls in a single aspirate then examine baby<br>Small residuals normal

**22.**	NICU	Bolus feeds	Not specified	Isolated large GRV in the absence of other clinical signs & symptoms should not prevent continued feeding<br>Signs of intolerance:<br>• Vomiting<br>• GRV >30% of previous 5 hours feed<br>• Abdo distention<br>• Unwell baby
**23.**	LNU	Bolus	4-6 hourly	If GRV 25-50% of total, replace the hourly amount, omit the feed and do not increase<br>If GRV >50% of total, stop feeds and medical review
**A.**	Network	Bolus	Not specified	GRV >25% (some >50%) in previous 4 hours in combination with vomiting and abdominal distention + bilious aspirates
**B.**	Network	Bolus feeds	4 hourly	Stop feeds if GRV heavily blood or bile stained<br>No mention of volume
**C.**	Network	Bolus feeds	Not specified	GRV should not be used in isolation to determine feed tolerance<br>Intolerance:<br>Vomiting + GRV >50% in the last 4 hours (especially if increasing) + abdominal distention
**D.**	Network	Bolus feeds advanced as per SIFT	Not specified	Infants 'feed tolerance' assessed with each set of cares (high risk), assess twice daily (mod risk) and before making changes in feed volumes (standard risk)<br>Assessing tolerance:<br>Undigested gastric residuals using a colour chart<br>GRV not used in isolation<br>But vomiting, GRV >25% of feed volume in last 4 hours + bloody or bilious residuals + abdominal distention
**E**	Network	Bolus	Assess GRV 4 - 6 hourly depending on cares	If GRV >50% of total, stop feeds and medical review<br>If GRV 25-50% of total, replace the hourly amount, omit the feed and do not increase<br>An appropriate GRV is <25% of preceding volume since last replacement of GRV<br>Replace GRV in full<br>A GRV >25% but <1.5mls unlikely to be problematic<br>A GRV of 25-50% is high, but acceptable if well, replace only normal hourly volume and continue feeds but do not increase<br>A GRV >50% is excessive, perform clinical exam, if acceptable hourly volume can be replaced but feed withheld

*Neonatal Unit Level determined by NNAP 2017 report (https://www.hqip.org.uk/resource/national-neonatal-audit-programme-2017-annual-report-on-2016-data/)

Abbreviations: GRV = Gastric Residual Volume; SIFT = Speed of Increasing of milk Feeds Trial(23), NICU = Neonatal Intensive Care Unit, LNU = Local Neonatal Unit, SCU = Special Care Unit.

BMJ Paediatrics Open

Gastric Residual Volume measurement in British neonatal intensive care units: a survey of practice

Journal:	*BMJ Paediatrics Open*
Manuscript ID	bmjpo-2019-000601.R1
Article Type:	Original research
Date Submitted by the Author:	14-May-2020
Complete List of Authors:	Dorling, Jon; Dalhousie University - Faculty of Medicine, Division of Neonatal-Perinatal Medicine<br>Tume, Lyvonne; University of Salford, Child Health<br>Arch, Barbara; University of Liverpool, Liverpool Clinical Trials Unit<br>Woolfall, Kerry; University of Liverpool, Health Services Research<br>Latten, Lynne; Alder Hey Children's Hospital<br>Roper, Louise; University of Liverpool, Department of Health Services Research<br>Deja, Elizabeth; University of Liverpool, Health Services Research<br>Pathan, Nazima; University of Cambridge, Paediatrics<br>Eccleson, Helen; University of Liverpool, Liverpool Clinical Trials Unit<br>Hickey, Helen; University of Liverpool, Liverpool Clinical Trials Unit<br>Brown, Michaela; University of Liverpool, Liverpool Clinical Trials Unit<br>Beissel, Anne; Hôpital Femme Mère Enfant, Neonatal Intensive Care Unit<br>Andrzejewska, izabela ; Chelsea and Westminster Healthcare NHS Trust, Neonatal Unit<br>Valla, Frederic; Hospices Civils de Lyon, Université Claude-Bernard Lyon 1, Service de réanimation pédiatrique, Hôpital Femme-Mère-Enfant<br>Gale, Chris; Imperial College London, Academic Neonatal Medicine
Keywords:	Measurement, Neonatology, Gastroenterology

SCHOLARONE™
Manuscripts

I, the Submitting Author has the right to grant and does grant on behalf of all authors of the Work (as defined in the below author licence), an exclusive licence and/or a non-exclusive licence for contributions from authors who are: i) UK Crown employees; ii) where BMJ has agreed a CC-BY licence shall apply, and/or iii) in accordance with the terms applicable for US Federal Government officers or employees acting as part of their official duties; on a worldwide, perpetual, irrevocable, royalty-free basis to BMJ Publishing Group Ltd ("BMJ") its licensees and where the relevant Journal is co-owned by BMJ to the co-owners of the Journal, to publish the Work in this journal and any other BMJ products and to exploit all rights, as set out in our licence.

The Submitting Author accepts and understands that any supply made under these terms is made by BMJ to the Submitting Author unless you are acting as an employee on behalf of your employer or a postgraduate student of an affiliated institution which is paying any applicable article publishing charge ("APC") for Open Access articles. Where the Submitting Author wishes to make the Work available on an Open Access basis (and intends to pay the relevant APC), the terms of reuse of such Open Access shall be governed by a Creative Commons licence – details of these licences and which Creative Commons licence will apply to this Work are set out in our licence referred to above.

Other than as permitted in any relevant BMJ Author's Self Archiving Policies, I confirm this Work has not been accepted for publication elsewhere, is not being considered for publication elsewhere and does not duplicate material already published. I confirm all authors consent to publication of this Work and authorise the granting of this licence.

Confidential: For Review Only

TITLE PAGE

Gastric Residual Volume measurement in British neonatal intensive care units: a survey of practice

Authors: Dorling J, Tume LN, Arch B, Woolfall K, Latten L, Roper L, Deja, E, Pathan N, Eccleson H,

Hickey H, Brown M, Beissel A, Andrzejewska I, Valla FV, Gale C

Corresponding author:

Jon Dorling FRCPCH, MD
Professor of Pediatrics and Division Head
Dalhousie University and IWK Health Centre
Division of Neonatal-Perinatal Medicine
5850/5980 University Avenue
P.O. Box 9700
Halifax, Nova Scotia, Canada, B3K 6R8
Jon.dorling@iwk.nshealth.ca

Lyvonne Tume, RN, PhD
Reader in Child Health,
University of Salford, Manchester UK
Frederick Road Campus, M6 6PU
Email: l.n.tume@salford.ac.uk

Barbara Arch MSc
Statistician
Liverpool Clinical Trials Unit
University of Liverpool
Institute in the Park
Alder Hey Children's NHS Foundation Trust
Liverpool
L12 2AP
barbara.arch@liverpool.ac.uk

Kerry Woolfall BA (Hons), MA, PhD
Senior Lecturer
Department of Health Services Research
University of Liverpool
Block B, Room B112, 1st Floor Waterhouse Building,
Liverpool L69 3GL
k.woolfall@liverpool.ac.uk

Lynne Latten Bsc (Hons), RD
Advanced Paediatric Dietitian Critical Care
Alder Hey Children's Hospital
Liverpool
L12 2AP
Lynne.Latten@alderhey.nhs.uk

https://mc.manuscriptcentral.com/bmjpo

Confidential: For Review Only

Louise Roper BSc, MSc, CPsychol, PhD
Chartered Health Psychologist,
Department of Health Services Research,
Block B, 1st Floor, Waterhouse Building,
University of Liverpool
Liverpool L69 3GX
Louise.Roper@liverpool.ac.uk

Elizabeth Deja BSc, MSc, PhD
Research Associate
Department of Health Services Research,
Block B, 1st Floor, Waterhouse Building,
University of Liverpool
Liverpool L69 3GX
bdeja1@liverpool.ac.uk

Nazima Pathan FRCPCH PhD
Consultant and University Lecturer in Paediatric Intensive Care
University of Cambridge
Addenbrooke's Hospital
Cambridge
CB2 0QQ
np409@cam.ac.uk

Helen Eccleson Bsc (Hons)
Trial Coordinator
Liverpool Clinical Trials Unit University of Liverpool
Institute in the Park
Alder Hey Children's NHS Foundation Trust
Liverpool
L12 2AP
Helen.Eccleson@liverpool.ac.uk

Helen Hickey PgD (Dist)
Head of Trial Management
Liverpool Clinical Trials Unit University of Liverpool
Institute in the Park
Alder Hey Children's NHS Foundation Trust
Liverpool
L12 2AP
Email: h.hickey@liverpool.ac.uk

Michaela Brown MSc
Senior Statistician
Liverpool Clinical Trials Unit
University of Liverpool
Institute in the Park
Alder Hey Children's NHS Foundation Trust
Liverpool
L12 2AP
Michaela.Brown@liverpool.ac.uk

Confidential: For Review Only

Anne Beissel MD
Consultant in Neonatal Intensive Care Unit
Neonatal Intensive Care Unit
Hôpital Femme Mère Enfant, Hospices Civils de Lyon
59 bd Pinel, 69500 Lyon-Bron, France
anne.beissel@chu-lyon.fr

Izabela Andrzejewska, RN MSc
Neonatal Unit Coordinator/Neonatal Research Nurse
Chelsea and Westminster Hospital
Neonatal Unit
3rd Floor, lift bank D
369 Fulham Road
London
SW10 9NH
i.andrzejewska@chelwest.nhs.uk

Frédéric V Valla MD MSc
Consultant in Pediatric Intensive Care Medicine
Pediatric Intensive Care Unit
CarMEN INSERM UMR 1060 Equipe INFOLIP
Hôpital Femme Mère Enfant, Hospices Civils de Lyon
59 bd Pinel, 69500 Lyon-Bron, France
Frederic.valla@chu-lyon.fr
and
Visiting Research Fellow
University of the West of England
Faculty of Health & Applied Sciences,
Blackberry Hill,
Bristol, BS16 1DD, UK

Chris Gale MRCPCH PhD
Reader in Neonatal Medicine
Imperial College London, Chelsea and Westminster Hospital campus,
London, SW10 9NH
Christopher.gale@imperial.ac.uk

This work was supported by NIHR HTA programme, grant number 16/94/02

Confidential: For Review Only

Dr. Dorling reports grants from National Institute for Health Research (NIHR), during the conduct of the study; grants from National Institute for Health Research, grants from Nutrinia, outside the submitted work.

Mrs. Latten reports grant from National Institute for Health Research, during the conduct of the study.

Dr. Deja reports grants from NIHR Health Technology Assessment Programme during the conduct of the study.

H. Eccleson reports grants from NIHR Health Technology Assessment during the conduct of the study.

H. Hickey reports this grant from NIHR Health Technology Assessment, during the conduct of the study.

M. Brown reports grants from NIHR HTA during the conduct of the study.

Dr. Valla reports personal fees from Baxter, personal fees from Nutricia, outside the submitted work.

Dr. Tume reports grants from NIHR during the conduct of the study.

Dr. Gale reports grants from National Institute for Health Research (NIHR), during the conduct of the study; grants from National Institute for Health Research, grants from Medical Research Foundation, grants from Mason Medical Research Foundation, grants and personal fees from Chiesi Pharmaceuticals, grants from Rosetrees Foundation, grants from Canadian Institute for Health Research, outside the submitted work

All other authors indicate that they have nothing to disclose.

All authors contributed to the study. JD and LT planned the study and incorporated comments from all members on the study design, conduct, interpretation and reporting of the work. BA analysed quantitative data, KW, ED and LR analysed the open answer responses, LT and JD analysed the guideline content. JD is responsible for the overall content as guarantor.

Confidential: For Review Only

Gastric Residual Volume measurement in British neonatal intensive care units: a survey of practice

Abstract

Objective: Despite little evidence, the practice of routine gastric residual volume (GRV) measurement to guide enteral feeding in neonatal units is widespread. Due to increased interest in this practice, and to examine trial feasibility, we aimed to determine enteral feeding and GRV measurement practices in British neonatal units.

Design & Setting: An online survey was distributed via email to all neonatal units and networks in England, Scotland and Wales. A clinical nurse, senior doctor and dietitian were invited to collaboratively complete the survey and submit a copy of relevant guidelines.

Results: 95/184 (51.6%) approached units completed the survey, 81/95 (85.3%) reported having feeding guidelines and 28 guidelines were submitted for review. The majority of units used intermittent (90/95) gastric feeds as their primary feeding method. 42/95 units reported specific guidance for measuring and interpreting GRV. 20/90 units measured GRV before every feed, 39/90 at regular time-intervals (most commonly 4-6 hourly 35/39) and 26/90 when felt to be clinically indicated. Most units reported uncertainty on the utility of aspirate volume for guiding feeding decisions; 13/90 reported that aspirate volume affected decisions 'very much'. In contrast, aspirate colour was reported to affect decisions 'very much' by 37/90 of responding units. Almost half, 44/90, routinely returned aspirates to the stomach.

Conclusions: Routine GRV measurement is part of standard practice in British neonatal units, although there was inconsistency in how frequently to measure or how to interpret the aspirate. Volume was considered less important than colour of the aspirate.

Keywords: newborn; infant; feeding; nutrition; feasibility study, questionnaire; critically ill

Confidential: For Review Only

INTRODUCTION

The gastric residual volume (GRV) is the volume of the entire stomach contents, obtained by aspiration with a syringe in order to assess feeding tolerance. It provides information on the volume and colour of fluid, and is distinct from the aspiration of a small amount of fluid for pH testing to confirm feeding tube position(1). There is a paucity of evidence to support routine measurement of GRV to direct and guide enteral feeding, and the practice is increasingly being questioned in neonatal units (1–5). For many clinicians, however, this parameter is a fundamental part of the definition and diagnosis of feed intolerance.(6) The rationale for routinely measuring GRV in the neonatal setting is for the early identification of necrotizing enterocolitis (NEC) and prevention of complications such as vomiting or aspiration, by withholding or reducing feed volumes. (1,7,8) Routine measurement, could, however, cause harm, for example through direct injury of the gastric mucosa, discarding gastric juices, medications and hormones, and by delaying enteral feeding and prolonging parenteral nutrition.(4,9,10) Furthermore, measurement of GRV has been shown to be inaccurate and affected by the position of the baby and the tube, hence it is not a useful surrogate marker for delayed gastric emptying in premature infants (11–14).

In this study, we aimed to identify current practice around GRV measurement in Great Britain. In addition, we sought to delineate enteral feeding practices in UK neonatal units in relation to GRV, and to identify a 'control arm' for a future trial comparing no routine GRV measurement (the intervention) to routine GRV measurement using this and work published elsewhere.

METHODS

A survey instrument was developed by the research team to explore current practices around GRV measurement and general enteral feeding practices in neonatal units. The intention was to use these survey findings alongside a review of neonatal unit guidelines to establish current practice. A 10 item closed question survey (tick-box responses) with optional free text response, and nine open-ended

Confidential: For Review Only

questions was developed by the researchers. The survey was piloted for face validity with 10 staff (doctors, dietitians, nurses). Minor wording adjustments were made to improve clarity, before the 19-item survey (Supplementary material) was entered onto the survey platform and retested by the study team.

The survey focussed on three domains: general enteral feeding and nutrition practices in the respondents' unit, the GRV measurement technique used in the respondents' unit, and clinical management in response to GRV. The survey invitation requested that a senior doctor, a clinical nurse and a dietitian complete the survey collaboratively and submit one response per unit, and requested that any relevant written guidelines or protocols be submitted. Unit name was collected, to target non-responders and check for duplicates; three reminders were sent to maximise response rates. Our target response rate was 70%.

All National Health Service (NHS) neonatal units in England, Scotland and Wales were approached during May and June 2018 using email invitations directed at 184 neonatal teams (some neonatal teams cover multiple neonatal units) sent through a national research collaboration, United Kingdom Neonatal Collaborative (UKNC), and a multi-disciplinary professional network, The Neonatal Nutrition Network (N3). Units in Northern Ireland were not contacted as they are not part of the UKNC. Study data were collected and managed using REDCap electronic data capture tools hosted at the University of Liverpool.(15) Data were summarised using descriptive statistics for quantitative data and a mix of thematic and content analysis for qualitative free text data.(16,17) Following this, the neonatal unit guidelines were reviewed and summarized. Ethical approval for the study was provided by the University of the West of England (Reference: HAS.18.04.144).
There was no direct patient or public involvement in the work presented in this manuscript as it involved surveying clinicians on their clinical practice. Other aspects of the research not reported

Confidential: For Review Only

here had substantial input as they involved qualitative interviews and consensus gathering (NIHR HTA journal in press).

RESULTS

95 of 184 (51.6%) neonatal units in the UK excluding Northern Ireland completed the survey. These consisted of 40 Neonatal Intensive Care Units (NICUs), 42 Local Neonatal Units (LNUs) and 13 Special Care Baby Units (SCBUs) giving response rates of 71.4%, 47.2% and 33.3% of the NICUs, LNUs and SCBUs respectively. Seventeen of a possible eighteen NICUs caring for both surgical and medical patients responded, as did 23 NICUs caring for medical cases only. LNUs and SCBUs do not provide early post-operative care in the UK.

Survey responses were received from senior doctors (81/95, 85.3%); nurses (51/95, 53.7%) and dietitians (9/95, 9.5%). Most (81/95, 85.3%) responding units reported written enteral feeding guidance and 28 unit or local neonatal network guidelines were sent to the author (Supplementary table). Enteral feeding was typically delivered intermittently (90/95, 94.7%) rather than continuously (5/95, 5.3%). 42/95 units (44.2%) reported having written guidance for measurement and interpretation of gastric residual volumes. Ninety units answered questions about the management of non-surgical babies. When asked about how often GRV is measured, 20/90 units (22.2%) measured aspirates before every feed, 26/90 (28.9%) when it was felt to be clinically indicated, and 39/90 (43.3%) measured GRV at regular time intervals (most commonly 4-6 hourly 35/39 (89.7%), but all more frequent than once per day). One unit had no guidelines on this, and 4/90 (4.4%) reported that they did not measure GRV. Among units that reported having written GRV measurement guidance, 13/39 (43.3%) indicated that the guidance was ‘always’, and 17/39 (38.6%) ‘usually’ followed, however free text responses suggested that practice was *“very variable depending on the nurse looking after the baby”* (Unit 3, surgical and medical unit). The bedside nurse

Confidential: For Review Only

most commonly made decisions in relation to GRV results, 56/90 (62.2%), followed by middle grade doctors, 41/90 (45.6%), and the senior nurse in charge of shift, 26/90 (28.9%).

Responding units had mixed views on how useful the volume of the aspirate was for guiding feeding decisions (Figure): just 13/90 (14.4%) of units reporting that volume affected clinical decision-making ‘very much’ and the most frequent response was an intermediate score. The colour of the aspirate was felt to be more important: 37/90 (41.1%) of units reporting that colour influenced clinical decisions ‘very much’ and this was the most frequent response. More detail was obtained from 74 open text responses to this question. A large volume of aspirate was commonly described as a concern, which would often lead to a clinical review of a baby’s condition and subsequent consideration of the how much milk the baby is receiving. The threshold for prompting a feeding review was reported to vary. Some units stated that aspirates over 50% of the feed would “prompt a review” (Unit 8, NICU surgical and medical), whilst others stated “>25% of feed given in previous 6 hours” (Unit 18, NICU medical only), if exceeds “25% of the previous 4 hours' feed volume” (Unit 22, NICU medical only) or “If >25% of the feed volume given since the last assessment was made” (Unit 25, NICU medical only).

Almost half, 44/90 (48.9%), routinely returned aspirates to the stomach. 72 nurses gave reasons for seeking medical advice: 55/72 (76.4%) cited increased or large volume GRVs, 52/72 (72.2%) cited bilious colour of the residual, or a change in colour. Other reasons were blood stained aspirates 16/72 (22.2%), concerns about condition of baby, such as desaturations 16/72 (22.2%), abdominal distention 11/72 (15.3%), and vomiting 5/72 (6.9%). In free text responses, units stated that that a dark or bilious colour would *“trigger medical review [by a] Middle Grade or Consultant”* (Unit 22, NICU medical only), whilst some described how feeds would be stopped: *“Green aspirate - assess baby and feeds withheld”* (Unit 60, LNU).

Confidential: For Review Only

Guideline analysis (Table 2 & Supplementary Table) revealed that 19 of 28 (67.8%) guidelines specified a volume of aspirate at which to consider stopping feeds using a defined proportion of the previous feed. Six guidelines specified this threshold as 25% or more of the previous feed, eight guidelines specified 50% or more, while five guidelines used a level between these. Fourteen guidelines mentioned the bilious green colouring of GRV being an indication to stop enteral feeds, while five mentioned blood staining as being important. Vomiting and abdominal distension were also considered important for guiding management being mentioned by 13 and 12 guidelines respectively.

DISCUSSION

The results of this survey confirm mixed practice in neonatal units across the United Kingdom for both monitoring GRV and in how findings are used to make decisions about enteral feeding. This survey also identifies that around half of British neonatal units use GRV as a parameter to guide enteral feeding advancement. Health professionals' views around the importance of the volume compared to the colour of the GRV were inconsistent and importance was defined at different thresholds. Aspirate colour was cited more often as important than volume of gastric residuals, however the importance of aspirate colour was inconsistent; some unit guidelines specified actions based on bilious or blood staining of the secretions whereas others did not mention them, and many unit guidelines referred to not returning aspirates that were bilious (green) or bloody (red) in colour. Change in aspirate colour was viewed as a potential indicator of NEC in preterm neonates in this survey, but this and many aspects of residual evaluation are unsubstantiated by high quality evidence.(5)

The mixed views elicited on interpreting volume are consistent with the paucity of evidence for routine GRV measurement, and support randomised trials to assess whether aspirating the stomach contents is a useful practice(4,7,9). Such a trial is a also supported by a recently

https://mc.manuscriptcentral.com/bmjpo

Confidential: For Review Only

published Cochrane review(1). Although it might be beneficial to stop measuring GRV in neonatal units, some health professionals believe their measurement can help to identify NEC earlier despite the absence of evidence to support this presumption. Recent results from small studies involving preterm infants suggests that not measuring GRV is not associated with an increase in the risk of NEC and might reduce the time to achieve full enteral feeds(3,4,9,18), however these studies were underpowered to detect even large relative differences in rare outcomes like NEC. Adequate power to definitively assess NEC would require a trial of thousands of participants rather than the 230 randomised participants studied to date(2,3). Routine monitoring of GRV does however add to nursing workload and may lead to other direct harms to the infant. Given the widespread use of this practice, a future trial would need to demonstrate the safety of both routinely monitoring and not monitoring GRV. Further details of the proposed trial are published elsewhere (NIHR HTA journal in press).

The routine measurement of GRV is based on the presumption that GRV are an accurate representation of the residual gastric contents. Laboratory-based simulation studies undermine this presumption, however, by demonstrating that GRV inaccurately measure gastric contents.(19,20) The GRV obtained is widely influenced by a number of factors such as the syringe size, gastric tube size and material, aspiration pressure, viscosity of aspirate, and both the position of the tube tip in the stomach and of the neonate.(21) Furthermore, when decision-making is based on volume, clinicians fail to consider the impact of gastric secretions produced during the digestion process.(22)

This study has limitations: firstly, as with any survey, responses may not reflect actual practice. However, we were able to obtain a summary of what ought to happen by reviewing unit guidelines. Secondly, it is a weakness of the study that there were low responses from the smaller neonatal units. The results might therefore over-represent the views of larger NICU units.

Confidential: For Review Only

CONCLUSIONS

The routine and frequent measurement of GRV is embedded in enteral feeding practice and guidelines in British neonatal units, despite a lack of evidence and questionable accuracy of this parameter. For many units, GRV is integral to the assessment of feed tolerance/intolerance with bilious colouring of the aspirate and presence of blood being considered important. This study has identified current practice around GRV measurement in British neonatal units, and supports examination of the benefits and harms of GRV in an adequately powered, randomised, controlled trial.

Acknowledgements: We thank all the neonatal units who took part in this survey.

Confidential: For Review Only

“What is already known on this topic” – followed by a maximum of 3 brief statements (no more than 25 words per statement);

1. The gastric residual volume is the volume of the entire stomach contents, obtained by aspiration with a syringe in order to assess feeding tolerance.

2. It is unclear if the routine measurement of gastric residual volume is beneficial or harmful in preterm infants.

3. Those who routinely measure GRV are attempting to identify necrotising enterocolitis early and aim to prevent complications by withholding or reducing feed volumes.

“What this study adds” – followed by a maximum of 3 brief statements (no more than 25 words per statement).

1. This study demonstrates mixed practice for residual measurements across neonatal units in Britain.
2. Aspirate colour was reported as affecting decisions more often in comparison to residual volume.
3. The heterogeneity of approaches regarding GRV measurement supports the need for a randomised trial to enable an evidence-based approach to the practice’

Confidential: For Review Only

REFERENCES

1. Abiramalatha T, Thanigainathan S, Ninan B. Routine monitoring of gastric residual for prevention of necrotising enterocolitis in preterm infants. Cochrane Database of Systematic Reviews 2019, Issue 7. Art. No.: CD012937. DOI: 10.1002/14651858.CD012937.pub2.

2. Singh B, Rochow N, Chessell L, Wilson J, Cunningham K, Fusch C, et al. Gastric Residual Volume in Feeding Advancement in Preterm Infants (GRIP Study): A Randomized Trial. J Pediatr. 2018;200:79-83.e1.

3. Parker LA, Weaver M, Torrazza RJM, Shuster J, Li N, Krueger C, et al. Effect of Gastric Residual Evaluation on Enteral Intake in Extremely Preterm Infants: A Randomized Clinical Trial. JAMA Pediatr. 2019 Jun 1;173(6):534–43.

4. Riskin A, Cohen K, Kugelman A, Toropine A, Said W, Bader D. The Impact of Routine Evaluation of Gastric Residual Volumes on the Time to Achieve Full Enteral Feeding in Preterm Infants. J Pediatr. 2017;189:128–34.

5. Li Y-F, Lin H-C, Torrazza RM, Parker L, Talaga E, Neu J. Gastric Residual Evaluation in Preterm Neonates: A Useful Monitoring Technique or a Hindrance? Pediatr Neonatol. 2014 Oct;55(5):335–40.

6. Moore TA, Wilson ME. Feeding Intolerance: A Concept Analysis. Adv Neonatal Care. 2011 Jun;11(3):149.

7. Cobb BA, Carlo WA, Ambalavanan N. Gastric Residuals and Their Relationship to Necrotizing Enterocolitis in Very Low Birth Weight Infants. Pediatrics. 2004 Jan 1;113(1):50–3.

8. Bertino E, Giuliani F, Prandi G, Coscia A, Martano C, Fabris C. Necrotizing enterocolitis: risk factor analysis and role of gastric residuals in very low birth weight infants. J Pediatr Gastroenterol Nutr. 2009 Apr;48(4):437–42.

9. Torrazza RM, Parker LA, Li Y, Talaga E, Shuster J, Neu J. The value of routine evaluation of gastric residuals in very low birth weight infants. J Perinatol Off J Calif Perinat Assoc. 2015 Jan;35(1):57–60.

10. Tume LN, Bickerdike A, Latten L, Davies S, Lefèvre MH, Nicolas GW, et al. Routine gastric residual volume measurement and energy target achievement in the PICU: a comparison study. Eur J Pediatr. 2017 Dec;176(12):1637–44.

11. Geraldo V, Pyati S, Joseph T, Pildes RS. Gastric Residual (GR): Reliability of the Measurement. 888. Pediatr Res. 1997 Apr 1;41(S4):150.

12. Parker L, Torrazza RM, Li Y, Talaga E, Shuster J, Neu J. Aspiration and evaluation of gastric residuals in the neonatal intensive care unit: state of the science. J Perinat Neonatal Nurs. 2015 Mar;29(1):51–9; quiz E2.

13. Shulman RJ, Ou C-N, Smith EO. Evaluation of potential factors predicting attainment of full gavage feedings in preterm infants. Neonatology. 2011;99(1):38–44.

https://mc.manuscriptcentral.com/bmjpo

Confidential: For Review Only

14. Jadcherla SR, Kliegman RM. Studies of feeding intolerance in very low birth weight infants: definition and significance. Pediatrics. 2002 Mar;109(3):516–7.

15. Harris PA, Taylor R, Thielke R, Payne J, Gonzalez N, Conde JG. Research electronic data capture (REDCap)--a metadata-driven methodology and workflow process for providing translational research informatics support. J Biomed Inform. 2009 Apr;42(2):377–81.

16. Hsieh H-F, Shannon SE. Three approaches to qualitative content analysis. Qual Health Res. 2005 Nov;15(9):1277–88.

17. Braun V, Clarke V. Using thematic analysis in psychology. Qual Res Psychol. 2006 Jan 1;3(2):77–101.

18. al BS et. Gastric Residual Volume in Feeding Advancement in Preterm Infants (GRIP Study): A Randomized Trial | Kopernio [Internet]. [cited 2019 Apr 25]. Available from: https://kopernio.com/viewer?doi=10.1016/j.jpeds.2018.04.072&token=WzExNzcyOCwiMTAuMTAxNi9qLmpwZWRzLjIwMTguMDQuMDcyIl0.58y70TAsh-b7Ky3TokWFqsMawr0

19. Bartlett Ellis RJ, Fuehne J. Examination of accuracy in the assessment of gastric residual volume: a simulated, controlled study. JPEN J Parenter Enteral Nutr. 2015 May;39(4):434–40.

20. Lin HC, Van Citters GW. Stopping enteral feeding for arbitrary gastric residual volume may not be physiologically sound: results of a computer simulation model. JPEN J Parenter Enteral Nutr. 1997 Oct;21(5):286–9.

21. Sangers H, Jong PM de, Mulder SE, Stigter GD, Berg CM van den, Pas AB te, et al. Outcomes of gastric residuals whilst feeding preterm infants in various body positions. J Neonatal Nurs. 2013 Dec 1;19(6):337–41.

22. Tume LN, Latten L, Kenworthy L. Paediatric intensive care nurses' decision-making around gastric residual volume measurement. Nurs Crit Care. 2017 Sep;22(5):293–7.

23. Abbott J, Berrington J, Bowler U, Boyle E, Dorling J, Embleton N, et al. The Speed of Increasing milk Feeds: a randomised controlled trial. BMC Pediatr. 2017 Jan 28;17:39.

Figure and Table legends

Figure: Perceived Importance of Aspirate Volume and Colour for making feeding decisions

Table 1: Survey results – General feeding practices for all babies

Table 2: Survey results - GRV practices specific to the management of medical babies

Supplementary Table: Detailed summary of British enteral feeding written guidelines

Confidential: For Review Only

Table 1: Survey results – General feeding practices for all babies (n=95)

Practice	N (%)
Units had written feeding guidelines/protocol	81 (85.3%)
Standard NG feeds were intermittent bolus (not continuous)	90 (94.7%)
There was specific guidance about how Gastric Residual Volume should be measured and interpreted - for example a protocol or guideline	42 (44.2%)
NICUs that care for surgical and medical babies (n=17):	
Gastric Residual Volume measurement differs between the medical and surgical babies	5/17 (29.4%)

Confidential: For Review Only

Table 2: Survey results - GRV practices specific to the management of medical babies (n=90)

Survey question	N (%)
**How often do staff in your unit measure GRV?**	
Once a day	0 (0%)
Before every feed	20 (22.2%)
Only when clinically indicated	26 (28.9%)
At regular intervals	39 (43.4%)
At least every 3,4 or 6 hrs	*35/39*
GRV is not measured	4 (4.4%)
**Is the specific guidance for GRV measurement followed and actually undertaken as per protocol – Only asked of units with specific guidance for GRV measurement (n=39)?**	
Always	13 (43.3%)
Usually	17 (38.6%)
Often	4 (10.3%)
Rarely / Never	5 (12.8%)
**Who usually decides what to do with concerning GRV aspirates in the first instance? (more than one response allowed)**	
Senior Doctor (Consultant)	13 (14.4%)
Middle Grade Doctor (SpR)	41 (45.6%)
Junior Grade Doctor (SHO)	18 (20.0%)
Bedside Nurse	56 (62.2%)
Nurse in charge of shift (senior nurse)	26 (28.9%)
**How much does volume of the aspirate affect your decision around GRV?**	
1 Not at all	5 (5.6%)
2	11 (12.2%)
3	40 (44.4%)
4	21 (23.3%)
5 (Very much)	13 (14.4%)
**How much does colour of the aspirate affect your decision around GRV?**	
1 Not at all	3 (3.3%)
2	6 (6.7%)
3	16 (17.8%)
4	28 (31.1%)
5 (Very much)	37 (41.1%)
**What do you do with obtained GRV: return or discard?**	
Return	44 (48.9%)
Discard	7 (7.8%)
Other	39 (43.3%)

Abbreviations: GRV Gastric Residual Volume

Confidential: For Review Only

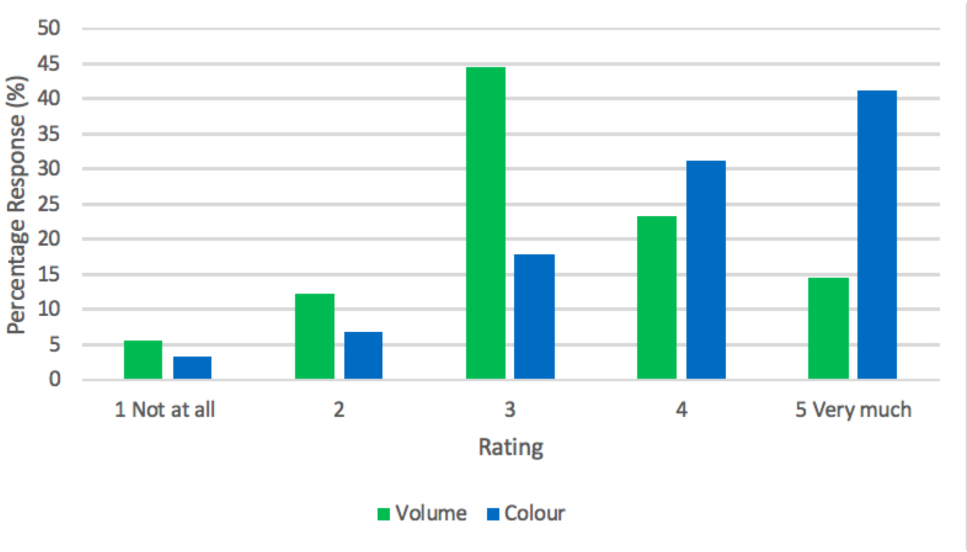
50
45
40
35
30
25
20
15
10
5
0
Percentage Response (%)
1 Not at all
2
3
4
5 Very much
Rating
Volume
Colour

Supplementary Table: Detailed summary of UK NU enteral feeding written guidelines

	Neonatal unit level*	Default feeding method	GRV checking	Threshold for stopping feeds
**1.**	LNU	Bolus feeds with advancement strategy as per SIFT trial	Not specifically mentioned	Aspirate >50% feed volume or green aspirates
**2.**	NICU	Bolus feeds	Routinely measured but no mention of frequency or technique	Aspirate >50% feed volume in previous 6 hours or bilious aspirates
**3.**	NICU	Bolus feeds	Measured but no mention of frequency or how	Aspirates >50% or >1ml<br>If aspirate contains blood or bile.<br>Discard GRV, stop feeds, wait 2 hours and re-assess
**4.**	NICU	Bolus	No mention of frequency or technique	Consider stopping if<br>• pre-feed aspirate >4mls/kg,<br>• heavy bile stained aspirates or<br>• 2 vomits after consecutive feeds
**5.**	LNU	Bolus with advancement as per SIFT trial	GRV aspirated 4 hourly	GRV >25% feeds in previous 4 hours combined with abdominal distention and/or vomiting
**6.**	NICU	No mention	No mention	No mention
**7.**	NICU	Bolus feeds and advanced as per SIFT trial	Check GRV no more than 6 hourly unless concerns	Withhold feeds for 6-12 hours if GRV >40% of feed given or 2 or 3ml (dependant on infant weight), heavily bile or blood stained or abdominal distention
**8.**	LNU	Not stated	Not stated	4 hourly NG aspirates are <25% of total infused in the preceding 4 hours<br>No significant abdominal distension<br>No significant vomiting<br>No bile -<br>stained aspirates
**9.**	NICU	Bolus feeds	Not stated	GRV >50% volume of feeds over last 6 hours or vomit of this size
**10.**	NICU	2 hourly bolus advanced as per SIFT	4-6 hourly	*Action with gastric residuals:*<br>If aspirates 25-50% of total, replace the volume, omit the feed and do not increment.<br>If aspirates >50% of total, stop feeds and medical review.<br>If dark bilious rather than lightly bile stained, stop feeds and medical review
**11.**	NICU	Bolus feeds, 2 risk levels, advanced as per SIFT	Routine measurement of full gastric residuals should be avoided. This should only be done, with discussion, as a part of<br>a full medical/ ANNP	Signs of feed intolerance may include clinical observations such as desaturation and bradycardia events and increased work of breathing, vomiting, abdominal distention and discolouration.
**12.**	SCU	Bolus feeds	Not specified	GRV > 2 hourly amount, vomiting or abdominal distention
**13.**	NICU	Bolus feeds	Not specified	**Medical babies:**<br>2ml/kg of milky gastric residual is not important and should simply be replaced. Where the<br>gastric residual at higher volumes is equivalent to 100% of the bolus, then the feeds should be<br>stopped and a clinical review<br>**Surgical babies:**<br>• aspirate <½ feed volume since last aspirate replace the aspirate itself and continue feeding

				• aspirate ≥ ½ feed volume but <whole feed volume replace half of the aspirate and discard the rest<br>• aspirate ≥whole feed volume since previous aspirate do not replace the aspirate, stop feeding & obtain senior medical and surgical review
**14.**	NICU	Bolus feeds	4 - 6 hourly	Examine and assess the baby if<br>• vomiting,<br>• GRV s >25% of the previous 4 hours total feed volume<br>• residuals are persisting or increasing<br>Small milky / yellow aspirates up to 2-3 mls are frequently normal. They can be replaced, and feeds continued
**15.**	LNU	Bolus feeds	Not specified	Aspirates up to 2-3ml or 50% of the previous 4 hours feed can be normal if the baby is well<br>Aspirates greater than 50% of the previous 4 hours feed or 2-3ml (whichever is greater) discard aspirate, hold feed and try again in 2 hours<br>If aspirate contains blood or bile then stop feeds
**16.**	NICU	Bolus feeds	4 - 6 h	When babies are on any enteral feeds, only aspirate the stomach contents via<br>a gastric tube every 4 - 6 hours, in order to check the residual volume. The assessment of the baby should include any abdominal distension, dark green (bilious) aspirates and bowel opening,<br><br>If <50% of the previous 4 - 6 hour total feed volume is aspirated, then replace the aspirate and continue enteral feeding, provided the baby is<br>otherwise clinically stable<br><br>If >50% of the previous 4 - 6 hour total feed volume is aspirated, then discuss with medical staff; often reasonable to replace the aspirate and omit the feed. If necessary, stop the feeds for 4 - 6 hours; a senior member of the medical / nursing team should then review
**17.**	NICU	Bolus feeds	Q 6 until infant is fully fed	Signs of intolerance<br>1. Vomiting<br>2. Gastric residuals >25% of previous 6<br>hours feed volume, persistent or increasing<br>3. Abdominal distension/increasing abdominal girth<br>4. Increase in stool frequency
**18.**	LNU	Bolus feeds	Not specified	If the aspirates are non-bilious and less than half the volume of previous feed they can be replaced and feeding continued while observing the infant closely<br><br>If the aspirates are bilious or >50% of the previous feed volume, consider withholding the feeds on that occasion and assess for any signs of NEC
**19.**	NICU	Bolus feeds	Not specified	Large volume aspirates or dark green bile stained aspirates, particularly in association with abdominal distension and/or tenderness are a cause for concern. Small milky / yellow aspirates up to 2-3 mls are frequently normal. They can be replaced, and feeds continued
**20.**	LNU	Bolus feeds	Not specified	No mention
**21.**	NICU	Bolus feeds	No more than 4 - 6h	If vomit or GRV exceed 33% of the last feed volume or are more than 3.5 mls in a single aspirate then examine baby<br>Small residuals normal

**22.**	NICU	Bolus feeds	Not specified	Isolated large GRV in the absence of other clinical signs & symptoms should not prevent continued feeding<br>Signs of intolerance:<br>• Vomiting<br>• GRV >30% of previous 5 hours feed<br>• Abdo distention<br>• Unwell baby
**23.**	LNU	Bolus	4-6 hourly	If GRV 25-50% of total, replace the hourly amount, omit the feed and do not increase<br>If GRV >50% of total, stop feeds and medical review
**A.**	Network	Bolus	Not specified	GRV >25% (some >50%) in previous 4 hours in combination with vomiting and abdominal distention + bilious aspirates
**B.**	Network	Bolus feeds	4 hourly	Stop feeds if GRV heavily blood or bile stained<br>No mention of volume
**C.**	Network	Bolus feeds	Not specified	GRV should not be used in isolation to determine feed tolerance<br>Intolerance:<br>Vomiting + GRV >50% in the last 4 hours (especially if increasing) + abdominal distention
**D.**	Network	Bolus feeds advanced as per SIFT	Not specified	Infants 'feed tolerance' assessed with each set of cares (high risk), assess twice daily (mod risk) and before making changes in feed volumes (standard risk)<br>Assessing tolerance:<br>Undigested gastric residuals using a colour chart<br>GRV not used in isolation<br>But vomiting, GRV >25% of feed volume in last 4 hours + bloody or bilious residuals + abdominal distention
**E**	Network	Bolus	Assess GRV 4 - 6 hourly depending on cares	If GRV >50% of total, stop feeds and medical review<br>If GRV 25-50% of total, replace the hourly amount, omit the feed and do not increase<br>An appropriate GRV is <25% of preceding volume since last replacement of GRV<br>Replace GRV in full<br>A GRV >25% but <1.5mls unlikely to be problematic<br>A GRV of 25-50% is high, but acceptable if well, replace only normal hourly volume and continue feeds but do not increase<br>A GRV >50% is excessive, perform clinical exam, if acceptable hourly volume can be replaced but feed withheld

*Neonatal Unit Level determined by NNAP 2017 report (https://www.hqip.org.uk/resource/national-neonatal-audit-programme-2017-annual-report-on-2016-data/)

Abbreviations: GRV = Gastric Residual Volume; SIFT = Speed of Increasing of milk Feeds Trial(23), NICU = Neonatal Intensive Care Unit, LNU = Local Neonatal Unit, SCU = Special Care Unit.

For peer review only

Introduction

We want to find out how Gastric Residual Volumes (GRV) are measured and used in your NICU. GRV measurement is aspirating ALL of the stomach contents to measure a 'volume' or 'aspirate' and often to look at the colour. This is NOT the small amount that is aspirated to test pH to confirm tube position.

We recommend a senior clinical nurse (who still does clinical practice) along with a senior doctor and/or a dietitian sit down together to complete this survey and we would like just one survey returned per NICU. If this is not possible, please respond without such a meeting.

Which neonatal unit do you work at?

Is the unit?

◯ NICU surgical and medical
◯ NICU medical only
◯ LNU
◯ SCBU

What are the job positions of the people completing this survey?

☐ Senior Doctor (Consultant)
☐ Middle Grade Doctor (SpR)
☐ Junior Grade Doctor (SHO)
☐ Nurse
☐ Dietitian

What band of nurse are you?

____

What band of dietitian are you?

____

Does your unit have written feeding guidelines/ protocol for medical and surgical babies?

◯ Yes
◯ No

We would greatly appreciate it if you would upload the feeding guidelines/protocol using the 'upload document' link below. Alternatively, they can be emailed to the GASTRIC study team at gastric.study@liverpool.ac.uk.

Are your standard NG feeds:

◯ Intermittent
◯ Continuous

Is there specific guidance about how Gastric Residual Volume should be measured and interpreted - for example a protocol or guideline?

◯ Yes
◯ No

We would greatly appreciate it if you would upload the guidelines/protocol using the 'upload document' link below. Alternatively, they can be emailed to the GASTRIC study team at gastric.study@liverpool.ac.uk.

Does Gastric Residual Volume measurement differ between the medical and surgical babies?

◯ Yes
◯ No

Please describe how it differs?

The rest of the survey is now ONLY asking about the management of preterm and term medical babies - not surgical babies.

How often do staff in your unit measure Gastric Residual Volume? (this question is NOT about aspirating a small amount to test pH, ONLY about how often you measure Gastric Residual Volume)

☐ Once a day
☐ Before every feed
☐ Only when clinically indicated
☐ Other

Please provide further details regarding how staff measure Gastric Residual Volume below; if 'other' has been selected, please also describe below:

Are Gastric Residual Volume measured for all babies, or just below a set gestational age/birth weight or for a specific condition?

Is the specific guidance for Gastric Residual Volume measurement followed and actually undertaken as per protocol? If needed, please ask a clinical member of your nursing team.

◯ Always
◯ Usually
◯ Often
◯ Rarely
◯ Never

Why is the guidance not always followed?

Who usually decides what to do with concerning Gastric Residual Volume aspirates in the first instance?

☐ Senior Doctor (Consultant)
☐ Middle Grade Doctor (SpR)
☐ Junior Grade Doctor (SHO)
☐ Bedside Nurse
☐ Nurse in charge of shift (senior nurse)

What band of nurse?

What would make a nurse seek advice from the medical team?

If you indicated that more than one person is involved in decision-making, please explain which factors may influence who makes the decision below:

How much does volume of the aspirate affect your decision around Gastric Residual Volume?

◯ 1 (Not at all) ◯ 2 ◯ 3 ◯ 4 ◯ 5 (Very much)

Please specify how:

How much does colour of the aspirate affect your decision around Gastric Residual Volume?

◯ 1 (Not at all) ◯ 2 ◯ 3 ◯ 4 ◯ 5 (Very much)

Please specify how:

What do you do with Gastric Residual Volume once obtained: return it or discard it?

◯ Return
◯ Discard
◯ Other

Please explain the factors that influence the decision to return or discard the Gastric Residual Volume. If 'other' selected, please also explain below:

Thank you for participating in the survey, your responses are greatly appreciated! If you have any further comments you wish to make, please provide below:

If you wish to be contacted about the study, please add your email address below (optional):

____

Only

BMJ Paediatrics Open

Gastric Residual Volume measurement in British neonatal intensive care units: a survey of practice

Journal:	*BMJ Paediatrics Open*
Manuscript ID	bmjpo-2019-000601.R2
Article Type:	Original research
Date Submitted by the Author:	04-Jun-2020
Complete List of Authors:	Dorling, Jon; Dalhousie University - Faculty of Medicine, Division of Neonatal-Perinatal Medicine<br>Tume, Lyvonne; University of Salford, Child Health<br>Arch, Barbara; University of Liverpool, Liverpool Clinical Trials Unit<br>Woolfall, Kerry; University of Liverpool, Health Services Research<br>Latten, Lynne; Alder Hey Children's Hospital<br>Roper, Louise; University of Liverpool, Department of Health Services Research<br>Deja, Elizabeth; University of Liverpool, Health Services Research<br>Pathan, Nazima; University of Cambridge, Paediatrics<br>Eccleson, Helen; University of Liverpool, Liverpool Clinical Trials Unit<br>Hickey, Helen; University of Liverpool, Liverpool Clinical Trials Unit<br>Brown, Michaela; University of Liverpool, Liverpool Clinical Trials Unit<br>Beissel, Anne; Hôpital Femme Mère Enfant, Neonatal Intensive Care Unit<br>Andrzejewska, izabela ; Chelsea and Westminster Healthcare NHS Trust, Neonatal Unit<br>Valla, Frederic; Hospices Civils de Lyon, Université Claude-Bernard Lyon 1, Service de réanimation pédiatrique, Hôpital Femme-Mère-Enfant<br>Gale, Chris; Imperial College London, Academic Neonatal Medicine
Keywords:	Measurement, Neonatology, Gastroenterology

SCHOLARONE™
Manuscripts

I, the Submitting Author has the right to grant and does grant on behalf of all authors of the Work (as defined in the below author licence), an exclusive licence and/or a non-exclusive licence for contributions from authors who are: i) UK Crown employees; ii) where BMJ has agreed a CC-BY licence shall apply, and/or iii) in accordance with the terms applicable for US Federal Government officers or employees acting as part of their official duties; on a worldwide, perpetual, irrevocable, royalty-free basis to BMJ Publishing Group Ltd ("BMJ") its licensees and where the relevant Journal is co-owned by BMJ to the co-owners of the Journal, to publish the Work in this journal and any other BMJ products and to exploit all rights, as set out in our licence.

The Submitting Author accepts and understands that any supply made under these terms is made by BMJ to the Submitting Author unless you are acting as an employee on behalf of your employer or a postgraduate student of an affiliated institution which is paying any applicable article publishing charge ("APC") for Open Access articles. Where the Submitting Author wishes to make the Work available on an Open Access basis (and intends to pay the relevant APC), the terms of reuse of such Open Access shall be governed by a Creative Commons licence – details of these licences and which Creative Commons licence will apply to this Work are set out in our licence referred to above.

Other than as permitted in any relevant BMJ Author's Self Archiving Policies, I confirm this Work has not been accepted for publication elsewhere, is not being considered for publication elsewhere and does not duplicate material already published. I confirm all authors consent to publication of this Work and authorise the granting of this licence.

Confidential: For Review Only

Confidential: For Review Only

TITLE PAGE

Gastric Residual Volume measurement in British neonatal intensive care units: a survey of practice

Authors: Dorling J, Tume LN, Arch B, Woolfall K, Latten L, Roper L, Deja, E, Pathan N, Eccleson H,

Hickey H, Brown M, Beissel A, Andrzejewska I, Valla FV, Gale C

Corresponding author:

Jon Dorling FRCPCH, MD
Professor of Pediatrics and Division Head
Dalhousie University and IWK Health Centre
Division of Neonatal-Perinatal Medicine
5850/5980 University Avenue
P.O. Box 9700
Halifax, Nova Scotia, Canada, B3K 6R8
Jon.dorling@iwk.nshealth.ca

Lyvonne Tume, RN, PhD
Reader in Child Health,
University of Salford, Manchester UK
Frederick Road Campus, M6 6PU
Email: l.n.tume@salford.ac.uk

Barbara Arch MSc
Statistician
Liverpool Clinical Trials Unit
University of Liverpool
Institute in the Park
Alder Hey Children's NHS Foundation Trust
Liverpool
L12 2AP
barbara.arch@liverpool.ac.uk

Kerry Woolfall BA (Hons), MA, PhD
Senior Lecturer
Department of Health Services Research
University of Liverpool
Block B, Room B112, 1st Floor Waterhouse Building,
Liverpool L69 3GL
k.woolfall@liverpool.ac.uk

Lynne Latten Bsc (Hons) RD
Advanced Paediatric Dietitian Critical Care
Alder Hey Children's Hospital
Liverpool
L12 2AP
Lynne.Latten@alderhey.nhs.uk

Louise Roper BSc, MSc, CPsychol, PhD
Chartered Health Psychologist,
Department of Health Services Research,
Block B, 1st Floor, Waterhouse Building,
University of Liverpool
Liverpool L69 3GX
Louise.Roper@liverpool.ac.uk

Elizabeth Deja BSc, MSc, PhD
Research Associate
Department of Health Services Research,
Block B, 1st Floor, Waterhouse Building,
University of Liverpool
Liverpool L69 3GX
bdeja1@liverpool.ac.uk

Nazima Pathan FRCPCH PhD
Consultant and University Lecturer in Paediatric Intensive Care
University of Cambridge
Addenbrooke's Hospital
Cambridge
CB2 0QQ
np409@cam.ac.uk

Helen Eccleson Bsc (Hons)
Trial Coordinator
Liverpool Clinical Trials Unit University of Liverpool
Institute in the Park
Alder Hey Children's NHS Foundation Trust
Liverpool
L12 2AP
Helen.Eccleson@liverpool.ac.uk

Helen Hickey PgD (Dist)
Head of Trial Management
Liverpool Clinical Trials Unit University of Liverpool
Institute in the Park
Alder Hey Children's NHS Foundation Trust
Liverpool
L12 2AP
Email: h.hickey@liverpool.ac.uk

Michaela Brown MSc
Senior Statistician
Liverpool Clinical Trials Unit
University of Liverpool
Institute in the Park
Alder Hey Children's NHS Foundation Trust
Liverpool
L12 2AP
Michaela.Brown@liverpool.ac.uk

Confidential: For Review Only

Anne Beissel MD
Consultant in Neonatal Intensive Care Unit
Neonatal Intensive Care Unit
Hôpital Femme Mère Enfant, Hospices Civils de Lyon
59 bd Pinel, 69500 Lyon-Bron, France
anne.beissel@chu-lyon.fr

Izabela Andrzejewska, RN MSc
Neonatal Unit Coordinator/Neonatal Research Nurse
Chelsea and Westminster Hospital
Neonatal Unit
3rd Floor, lift bank D
369 Fulham Road
London
SW10 9NH
i.andrzejewska@chelwest.nhs.uk

Frédéric V Valla MD MSc
Consultant in Pediatric Intensive Care Medicine
Pediatric Intensive Care Unit
CarMEN INSERM UMR 1060 Equipe INFOLIP
Hôpital Femme Mère Enfant, Hospices Civils de Lyon
59 bd Pinel, 69500 Lyon-Bron, France
Frederic.valla@chu-lyon.fr
and
Visiting Research Fellow
University of the West of England
Faculty of Health & Applied Sciences,
Blackberry Hill,
Bristol, BS16 1DD, UK

Chris Gale MRCPCH PhD
Reader in Neonatal Medicine
Imperial College London, Chelsea and Westminster Hospital campus,
London, SW10 9NH
Christopher.gale@imperial.ac.uk

This work was supported by NIHR HTA programme as part of a larger NIHR HTA–funded feasibility study, grant number 16/94/02. The views expressed are those of the authors and not necessarily those of the NHS, the NIHR, or the Department of Health and Social Care.

For Review Only

Dr. Dorling reports grants from National Institute for Health Research (NIHR), during the conduct of the study; grants from National Institute for Health Research, grants from Nutrinia, outside the submitted work.

Mrs. Latten reports grant from National Institute for Health Research, during the conduct of the study.

Dr. Deja reports grants from NIHR Health Technology Assessment Programme during the conduct of the study.

H. Eccleson reports grants from NIHR Health Technology Assessment during the conduct of the study.

H. Hickey reports this grant from NIHR Health Technology Assessment, during the conduct of the study.

M. Brown reports grants from NIHR HTA during the conduct of the study.

Dr. Valla reports personal fees from Baxter, personal fees from Nutricia, outside the submitted work.

Dr. Tume reports grants from NIHR during the conduct of the study.

Dr. Gale reports grants from National Institute for Health Research (NIHR), during the conduct of the study; grants from National Institute for Health Research, grants from Medical Research Foundation, grants from Mason Medical Research Foundation, grants and personal fees from Chiesi Pharmaceuticals, grants from Rosetrees Foundation, grants from Canadian Institute for Health Research, outside the submitted work

All other authors indicate that they have nothing to disclose.

All authors contributed to the study. JD and LT planned the study and incorporated comments from all members on the study design, conduct, interpretation and reporting of the work. BA analysed quantitative data, KW, ED and LR analysed the open answer responses, LT and JD analysed the guideline content. JD is responsible for the overall content as guarantor.

Confidential: For Review Only

Gastric Residual Volume measurement in British neonatal intensive care units: a survey of practice

Abstract

Objective: Despite little evidence, the practice of routine gastric residual volume (GRV) measurement to guide enteral feeding in neonatal units is widespread. Due to increased interest in this practice, and to examine trial feasibility, we aimed to determine enteral feeding and GRV measurement practices in British neonatal units.

Design & Setting: An online survey was distributed via email to all neonatal units and networks in England, Scotland and Wales. A clinical nurse, senior doctor and dietitian were invited to collaboratively complete the survey and submit a copy of relevant guidelines.

Results: 95/184 (51.6%) approached units completed the survey, 81/95 (85.3%) reported having feeding guidelines and 28 guidelines were submitted for review. The majority of units used intermittent (90/95) gastric feeds as their primary feeding method. 42/95 units reported specific guidance for measuring and interpreting GRV. 20/90 units measured GRV before every feed, 39/90 at regular time-intervals (most commonly 4-6 hourly 35/39) and 26/90 when felt to be clinically indicated. Most units reported uncertainty on the utility of aspirate volume for guiding feeding decisions; 13/90 reported that aspirate volume affected decisions 'very much'. In contrast, aspirate colour was reported to affect decisions 'very much' by 37/90 of responding units. Almost half, 44/90, routinely returned aspirates to the stomach.

Conclusions: Routine GRV measurement is part of standard practice in British neonatal units, although there was inconsistency in how frequently to measure or how to interpret the aspirate. Volume was considered less important than colour of the aspirate.

Keywords: newborn; infant; feeding; nutrition; feasibility study, questionnaire; critically ill

Confidential: For Review Only

INTRODUCTION

The gastric residual volume (GRV) is the volume of the entire stomach contents, obtained by aspiration with a syringe in order to assess feeding tolerance. It provides information on the volume and colour of fluid, and is distinct from the aspiration of a small amount of fluid for pH testing to confirm feeding tube position(1). There is a paucity of evidence to support routine measurement of GRV to direct and guide enteral feeding, and the practice is increasingly being questioned in neonatal units (1–5). For many clinicians, however, this parameter is a fundamental part of the definition and diagnosis of feed intolerance.(6) The rationale for routinely measuring GRV in the neonatal setting is for the early identification of necrotizing enterocolitis (NEC) and prevention of complications such as vomiting or aspiration, by withholding or reducing feed volumes. (1,7,8) Routine measurement, could, however, cause harm, for example through direct injury of the gastric mucosa, discarding gastric juices, medications and hormones, and by delaying enteral feeding and prolonging parenteral nutrition.(4,9,10) Furthermore, measurement of GRV has been shown to be inaccurate and affected by the position of the baby and the tube, hence it is not a useful surrogate marker for delayed gastric emptying in premature infants (11–14).

In this study, we aimed to identify current practice around GRV measurement in Great Britain. In addition, we sought to delineate enteral feeding practices in UK neonatal units in relation to GRV, and to identify a 'control arm' for a future trial comparing no routine GRV measurement (the intervention) to routine GRV measurement using this and work published elsewhere.

METHODS

A survey instrument was developed by the research team to explore current practices around GRV measurement and general enteral feeding practices in neonatal units. The intention was to use these survey findings alongside a review of neonatal unit guidelines to establish current practice. A 10 item closed question survey (tick-box responses) with optional free text response, and nine open-ended

Confidential: For Review Only

questions was developed by the researchers. The survey was piloted for face validity with 10 staff (doctors, dietitians, nurses). Minor wording adjustments were made to improve clarity, before the 19-item survey (Supplementary material) was entered onto the survey platform and retested by the study team.

The survey focussed on three domains: general enteral feeding and nutrition practices in the respondents' unit, the GRV measurement technique used in the respondents' unit, and clinical management in response to GRV. The survey invitation requested that a senior doctor, a clinical nurse and a dietitian complete the survey collaboratively and submit one response per unit, and requested that any relevant written guidelines or protocols be submitted. Unit name was collected, to target non-responders and check for duplicates; three reminders were sent to maximise response rates. Our target response rate was 70%.

All National Health Service (NHS) neonatal units in England, Scotland and Wales were approached during May and June 2018 using email invitations directed at 184 neonatal teams (some neonatal teams cover multiple neonatal units) sent through a national research collaboration, United Kingdom Neonatal Collaborative (UKNC), and a multi-disciplinary professional network, The Neonatal Nutrition Network (N3). Units in Northern Ireland were not contacted as they are not part of the UKNC. Study data were collected and managed using REDCap electronic data capture tools hosted at the University of Liverpool.(15) Data were summarised using descriptive statistics for quantitative data and a mix of thematic and content analysis for qualitative free text data.(16,17) Following this, the neonatal unit guidelines were reviewed and summarized. Ethical approval for the study was provided by the University of the West of England (Reference: HAS.18.04.144). There was no direct patient or public involvement in the work presented in this manuscript as it involved surveying clinicians on their clinical practice. Other aspects of the research not reported here had substantial input as they involved qualitative interviews and consensus gathering (18).

Confidential: For Review Only

RESULTS

95 of 184 (51.6%) neonatal units in the UK excluding Northern Ireland completed the survey. These consisted of 40 Neonatal Intensive Care Units (NICUs), 42 Local Neonatal Units (LNUs) and 13 Special Care Baby Units (SCBUs) giving response rates of 71.4%, 47.2% and 33.3% of the NICUs, LNUs and SCBUs respectively. Seventeen of a possible eighteen NICUs caring for both surgical and medical patients responded, as did 23 NICUs caring for medical cases only. LNUs and SCBUs do not provide early post-operative care in the UK.

Survey responses were received from senior doctors (81/95, 85.3%); nurses (51/95, 53.7%) and dietitians (9/95, 9.5%). Most (81/95, 85.3%) responding units reported written enteral feeding guidance and 28 unit or local neonatal network guidelines were sent to the author (Supplementary table 2). Enteral feeding was typically delivered intermittently (90/95, 94.7%) rather than continuously (5/95, 5.3%). 42/95 units (44.2%) reported having written guidance for measurement and interpretation of gastric residual volumes. Ninety units answered questions about the management of non-surgical babies. When asked about how often GRV is measured, 20/90 units (22.2%) measured aspirates before every feed, 26/90 (28.9%) when it was felt to be clinically indicated, and 39/90 (43.3%) measured GRV at regular time intervals (most commonly 4-6 hourly 35/39 (89.7%), but all more frequent than once per day). One unit had no guidelines on this, and 4/90 (4.4%) reported that they did not measure GRV. 90 open text responses were received to the question "Are Gastric Residual Volume measured for all babies, or just below a set gestational age/birth weight or for a specific condition"? Over 30 responses said all babies should have gastric aspirates measured, with some additional responses limiting this to those on gastric tube feeds or until full feeds are established. Just six responses mentioned a gestational age cut off, four suggesting <32 weeks, one <27 weeks and one <34 weeks gestation. Just one response indicated a birthweight criterion (under 1500g at birth). Supplementary table 1 presents the responses.

https://mc.manuscriptcentral.com/bmjpo

Among units that reported having written GRV measurement guidance, 13/39 (43.3%) indicated that the guidance was 'always', and 17/39 (38.6%) 'usually' followed, however free text responses suggested that practice was *"very variable depending on the nurse looking after the baby"* (Unit 3, surgical and medical unit). The bedside nurse most commonly made decisions in relation to GRV results, 56/90 (62.2%), followed by middle grade doctors, 41/90 (45.6%), and the senior nurse in charge of shift, 26/90 (28.9%).

Responding units had mixed views on how useful the volume of the aspirate was for guiding feeding decisions (Figure): just 13/90 (14.4%) of units reporting that volume affected clinical decision-making 'very much' and the most frequent response was an intermediate score. The colour of the aspirate was felt to be more important: 37/90 (41.1%) of units reporting that colour influenced clinical decisions 'very much' and this was the most frequent response. More detail was obtained from 74 open text responses to this question. A large volume of aspirate was commonly described as a concern, which would often lead to a clinical review of a baby's condition and subsequent consideration of the how much milk the baby is receiving. The threshold for prompting a feeding review was reported to vary. Some units stated that aspirates over 50% of the feed would "prompt a review" (Unit 8, NICU surgical and medical), whilst others stated ">25% of feed given in previous 6 hours" (Unit 18, NICU medical only), if exceeds "25% of the previous 4 hours' feed volume" (Unit 22, NICU medical only) or "If >25% of the feed volume given since the last assessment was made" (Unit 25, NICU medical only).

Almost half, 44/90 (48.9%), routinely returned aspirates to the stomach. 72 nurses gave reasons for seeking medical advice: 55/72 (76.4%) cited increased or large volume GRVs, 52/72 (72.2%) cited bilious colour of the residual, or a change in colour. Other reasons were blood stained aspirates 16/72 (22.2%), concerns about condition of baby, such as desaturations 16/72 (22.2%), abdominal

Confidential: For Review Only

distention 11/72 (15.3%), and vomiting 5/72 (6.9%). In free text responses, units stated that that a dark or bilious colour would *"trigger medical review [by a] Middle Grade or Consultant"* (Unit 22, NICU medical only), whilst some described how feeds would be stopped: *"Green aspirate - assess baby and feeds withheld"* (Unit 60, LNU).

Guideline analysis (Table 2 & Supplementary Table 2) revealed that 19 of 28 (67.8%) guidelines specified a volume of aspirate at which to consider stopping feeds using a defined proportion of the previous feed. Six guidelines specified this threshold as 25% or more of the previous feed, eight guidelines specified 50% or more, while five guidelines used a level between these. Fourteen guidelines mentioned the bilious green colouring of GRV being an indication to stop enteral feeds, while five mentioned blood staining as being important. Vomiting and abdominal distension were also considered important for guiding management being mentioned by 13 and 12 guidelines respectively.

DISCUSSION

The results of this survey confirm mixed practice in neonatal units across the United Kingdom for both monitoring GRV and in how findings are used to make decisions about enteral feeding. This survey also identifies that around half of British neonatal units use GRV as a parameter to guide enteral feeding advancement. Health professionals' views around the importance of the volume compared to the colour of the GRV were inconsistent and importance was defined at different thresholds. Aspirate colour was cited more often as important than volume of gastric residuals, however the importance of aspirate colour was inconsistent; some unit guidelines specified actions based on bilious or blood staining of the secretions whereas others did not mention them, and many unit guidelines referred to not returning aspirates that were bilious (green) or bloody (red) in colour. Change in aspirate colour was viewed as a potential indicator of NEC in preterm neonates

Confidential: For Review Only

in this survey, but this and many aspects of residual evaluation are unsubstantiated by high quality evidence.(5)

The mixed views elicited on interpreting volume are consistent with the paucity of evidence for routine GRV measurement, and support randomised trials to assess whether aspirating the stomach contents is a useful practice(4,7,9). Such a trial is a also supported by a recently published Cochrane review(1). Although it might be beneficial to stop measuring GRV in neonatal units, some health professionals believe their measurement can help to identify NEC earlier despite the absence of evidence to support this presumption. Recent results from small studies involving preterm infants suggests that not measuring GRV is not associated with an increase in the risk of NEC and might reduce the time to achieve full enteral feeds(3,4,9,19), however these studies were underpowered to detect even large relative differences in rare outcomes like NEC. Adequate power to definitively assess NEC would require a trial of thousands of participants rather than the 230 randomised participants studied to date(2,3). Routine monitoring of GRV does however add to nursing workload and may lead to other direct harms to the infant. Given the widespread use of this practice, a future trial would need to demonstrate the safety of both routinely monitoring and not monitoring GRV. Further details of the proposed trial are published elsewhere (NIHR HTA journal in press).

The routine measurement of GRV is based on the presumption that GRV are an accurate representation of the residual gastric contents. Laboratory-based simulation studies undermine this presumption, however, by demonstrating that GRV inaccurately measure gastric contents.(20,21) The GRV obtained is widely influenced by a number of factors such as the syringe size, gastric tube size and material, aspiration pressure, viscosity of aspirate, and both the position of the tube tip in the stomach and of the neonate.(22) Furthermore, when decision-making is

Confidential: For Review Only

based on volume, clinicians fail to consider the impact of gastric secretions produced during the digestion process.(23)

This study has limitations: firstly, as with any survey, responses may not reflect actual practice. However, we were able to obtain a summary of what ought to happen by reviewing unit guidelines. Secondly, it is a weakness of the study that there were low responses from the smaller neonatal units. The results might therefore over-represent the views of larger NICU units. Thirdly, we asked an open rather than a closed question to seek detail on which babies (in terms of gestation, birthweight or conditions) have residual volumes measured which made the data hard to analyse. Further details were obtained in related research and have been published elsewhere (NIHR HTA Journals in press)

CONCLUSIONS

The routine and frequent measurement of GRV is embedded in enteral feeding practice and guidelines in British neonatal units, despite a lack of evidence and questionable accuracy of this parameter. For many units, GRV is integral to the assessment of feed tolerance/intolerance with bilious colouring of the aspirate and presence of blood being considered important. This study has identified current practice around GRV measurement in British neonatal units, and supports examination of the benefits and harms of GRV in an adequately powered, randomised, controlled trial.

Acknowledgements: We thank all the neonatal units who took part in this survey.

This publication contains information also published in the NIHR HTA Journal under an agreement that this acknowledgement is made.(18)

Confidential: For Review Only

"What is already known on this topic" – followed by a maximum of 3 brief statements (no more than 25 words per statement);

1. The gastric residual volume is the volume of the entire stomach contents, obtained by aspiration with a syringe in order to assess feeding tolerance.

2. It is unclear if the routine measurement of gastric residual volume is beneficial or harmful in preterm infants.

3. Those who routinely measure GRV are attempting to identify necrotising enterocolitis early and aim to prevent complications by withholding or reducing feed volumes.

"What this study adds" – followed by a maximum of 3 brief statements (no more than 25 words per statement).

1. This study demonstrates mixed practice for residual measurements across neonatal units in Britain.
2. Aspirate colour was reported as affecting decisions more often in comparison to residual volume.
3. A randomised trial appears feasible in Great Britain given the variation in practice and willingness of respondents to randomise to measuring or not measuring.

Confidential: For Review Only

REFERENCES

1. Mihatsch WA, von Schoenaich P, Fahnenstich H, Dehne N, Ebbecke H, Plath C, et al. The Significance of Gastric Residuals in the Early Enteral Feeding Advancement of Extremely Low Birth Weight Infants. Pediatrics. 2002 Mar;109(3):457–459.

2. Singh B, Rochow N, Chessell L, Wilson J, Cunningham K, Fusch C, et al. Gastric Residual Volume in Feeding Advancement in Preterm Infants (GRIP Study): A Randomized Trial. J Pediatr. 2018;200:79-83.e1.

3. Parker LA, Weaver M, Torrazza RJM, Shuster J, Li N, Krueger C, et al. Effect of Gastric Residual Evaluation on Enteral Intake in Extremely Preterm Infants: A Randomized Clinical Trial. JAMA Pediatr. 2019 Jun 1;173(6):534–43.

4. Riskin A, Cohen K, Kugelman A, Toropine A, Said W, Bader D. The Impact of Routine Evaluation of Gastric Residual Volumes on the Time to Achieve Full Enteral Feeding in Preterm Infants. J Pediatr. 2017;189:128–34.

5. Li Y-F, Lin H-C, Torrazza RM, Parker L, Talaga E, Neu J. Gastric Residual Evaluation in Preterm Neonates: A Useful Monitoring Technique or a Hindrance? Pediatr Neonatol. 2014 Oct;55(5):335–40.

6. Moore TA, Wilson ME. Feeding Intolerance: A Concept Analysis. Adv Neonatal Care. 2011 Jun;11(3):149.

7. Cobb BA, Carlo WA, Ambalavanan N. Gastric Residuals and Their Relationship to Necrotizing Enterocolitis in Very Low Birth Weight Infants. Pediatrics. 2004 Jan 1;113(1):50–3.

8. Bertino E, Giuliani F, Prandi G, Coscia A, Martano C, Fabris C. Necrotizing enterocolitis: risk factor analysis and role of gastric residuals in very low birth weight infants. J Pediatr Gastroenterol Nutr. 2009 Apr;48(4):437–42.

9. Torrazza RM, Parker LA, Li Y, Talaga E, Shuster J, Neu J. The value of routine evaluation of gastric residuals in very low birth weight infants. J Perinatol Off J Calif Perinat Assoc. 2015 Jan;35(1):57–60.

10. Tume LN, Bickerdike A, Latten L, Davies S, Lefèvre MH, Nicolas GW, et al. Routine gastric residual volume measurement and energy target achievement in the PICU: a comparison study. Eur J Pediatr. 2017 Dec;176(12):1637–44.

11. Geraldo V, Pyati S, Joseph T, Pildes RS. Gastric Residual (GR): Reliability of the Measurement. 888. Pediatr Res. 1997 Apr 1;41(S4):150.

12. Parker L, Torrazza RM, Li Y, Talaga E, Shuster J, Neu J. Aspiration and evaluation of gastric residuals in the neonatal intensive care unit: state of the science. J Perinat Neonatal Nurs. 2015 Mar;29(1):51–9; quiz E2.

13. Shulman RJ, Ou C-N, Smith EO. Evaluation of potential factors predicting attainment of full gavage feedings in preterm infants. Neonatology. 2011;99(1):38–44.

Confidential: For Review Only

14. Jadcherla SR, Kliegman RM. Studies of feeding intolerance in very low birth weight infants: definition and significance. Pediatrics. 2002 Mar;109(3):516–7.

15. Harris PA, Taylor R, Thielke R, Payne J, Gonzalez N, Conde JG. Research electronic data capture (REDCap)--a metadata-driven methodology and workflow process for providing translational research informatics support. J Biomed Inform. 2009 Apr;42(2):377–81.

16. Hsieh H-F, Shannon SE. Three approaches to qualitative content analysis. Qual Health Res. 2005 Nov;15(9):1277–88.

17. Braun V, Clarke V. Using thematic analysis in psychology. Qual Res Psychol. 2006 Jan 1;3(2):77–101.

18. Ln T, K W, B A, L R, E D, Ap J, et al. Routine gastric residual volume measurement to guide enteral feeding in mechanically ventilated infants and children: the GASTRIC feasibility study. Health Technol Assess [Internet]. 2020 May 27 [cited 2020 Jun 4];24(23). Available from: https://www.journalslibrary.nihr.ac.uk/hta/hta24230#/abstract

19. al BS et. Gastric Residual Volume in Feeding Advancement in Preterm Infants (GRIP Study): A Randomized Trial | Kopernio [Internet]. [cited 2019 Apr 25]. Available from: https://kopernio.com/viewer?doi=10.1016/j.jpeds.2018.04.072&token=WzExNzcyOCwiMTAuMTAxNi9qLmpwZWRzLjIwMTguMDQuMDcyIl0.58y70TAsh-b7Ky3TokWFqsMawr0

20. Bartlett Ellis RJ, Fuehne J. Examination of accuracy in the assessment of gastric residual volume: a simulated, controlled study. JPEN J Parenter Enteral Nutr. 2015 May;39(4):434–40.

21. Lin HC, Van Citters GW. Stopping enteral feeding for arbitrary gastric residual volume may not be physiologically sound: results of a computer simulation model. JPEN J Parenter Enteral Nutr. 1997 Oct;21(5):286–9.

22. Sangers H, Jong PM de, Mulder SE, Stigter GD, Berg CM van den, Pas AB te, et al. Outcomes of gastric residuals whilst feeding preterm infants in various body positions. J Neonatal Nurs. 2013 Dec 1;19(6):337–41.

23. Tume LN, Latten L, Kenworthy L. Paediatric intensive care nurses' decision-making around gastric residual volume measurement. Nurs Crit Care. 2017 Sep;22(5):293–7.

Figure and Table legends

Figure: Perceived Importance of Aspirate Volume and Colour for making feeding decisions

Table 1: Survey results – General feeding practices for all babies

Table 2: Survey results - GRV practices specific to the management of medical babies

Confidential: For Review Only

Supplementary Table 1: Individual responses to the question "Are Gastric Residual Volume measured for all babies, or just below a set gestational age/birth weight or for a specific condition"?

Supplementary Table 2: Detailed summary of British enteral feeding written guidelines

Confidential: For Review Only

Table 1: Survey results – General feeding practices for all babies (n=95)

Practice	N (%)
Units had written feeding guidelines/protocol	81 (85.3%)
Standard NG feeds were intermittent bolus (not continuous)	90 (94.7%)
There was specific guidance about how Gastric Residual Volume should be measured and interpreted - for example a protocol or guideline	42 (44.2%)
NICUs that care for surgical and medical babies (n=17):	
Gastric Residual Volume measurement differs between the medical and surgical babies	5/17 (29.4%)

Table 2: Survey results - GRV practices specific to the management of medical babies (n=90)

Survey question	N (%)
**How often do staff in your unit measure GRV?**	
Once a day	0 (0%)
Before every feed	20 (22.2%)
Only when clinically indicated	26 (28.9%)
At regular intervals	39 (43.4%)
At least every 3,4 or 6 hrs	*35/39*
GRV is not measured	4 (4.4%)
**Is the specific guidance for GRV measurement followed and actually undertaken as per protocol – Only asked of units with specific guidance for GRV measurement (n=39)?**	
Always	13 (43.3%)
Usually	17 (38.6%)
Often	4 (10.3%)
Rarely / Never	5 (12.8%)
**Who usually decides what to do with concerning GRV aspirates in the first instance? (more than one response allowed)**	
Senior Doctor (Consultant)	13 (14.4%)
Middle Grade Doctor (SpR)	41 (45.6%)
Junior Grade Doctor (SHO)	18 (20.0%)
Bedside Nurse	56 (62.2%)
Nurse in charge of shift (senior nurse)	26 (28.9%)
**How much does volume of the aspirate affect your decision around GRV?**	
1 Not at all	5 (5.6%)
2	11 (12.2%)
3	40 (44.4%)
4	21 (23.3%)
5 (Very much)	13 (14.4%)
**How much does colour of the aspirate affect your decision around GRV?**	
1 Not at all	3 (3.3%)
2	6 (6.7%)
3	16 (17.8%)
4	28 (31.1%)
5 (Very much)	37 (41.1%)
**What do you do with obtained GRV: return or discard?**	
Return	44 (48.9%)
Discard	7 (7.8%)
Other	39 (43.3%)

Abbreviations: GRV Gastric Residual Volume

Confidential: For Review Only

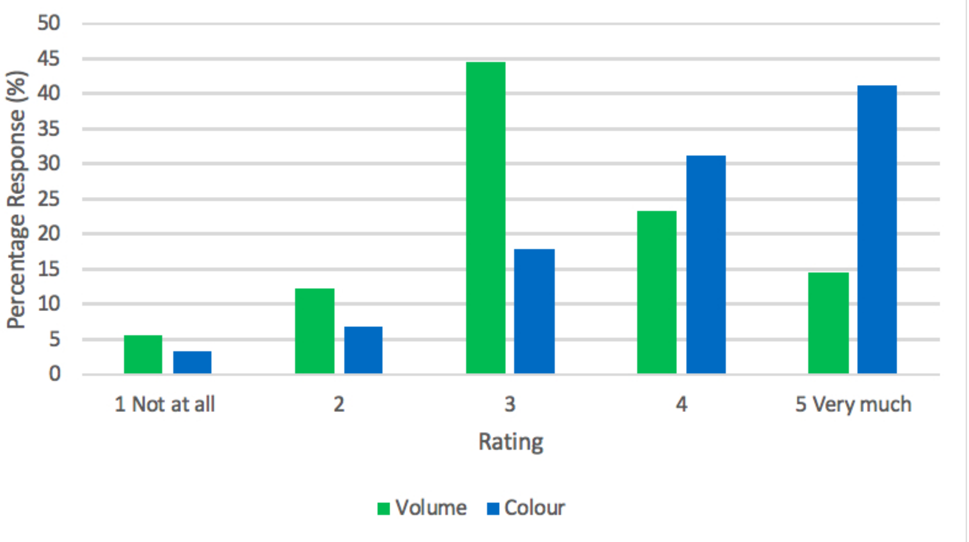
50
45
40
35
30
25
20
15
10
5
0
Percentage Response (%)
1 Not at all
2
3
4
5 Very much
Rating
Volume
Colour

Introduction

We want to find out how Gastric Residual Volumes (GRV) are measured and used in your NICU. GRV measurement is aspirating ALL of the stomach contents to measure a 'volume' or 'aspirate' and often to look at the colour. This is NOT the small amount that is aspirated to test pH to confirm tube position.

We recommend a senior clinical nurse (who still does clinical practice) along with a senior doctor and/or a dietitian sit down together to complete this survey and we would like just one survey returned per NICU. If this is not possible, please respond without such a meeting.

Which neonatal unit do you work at?

Is the unit?

◯ NICU surgical and medical
◯ NICU medical only
◯ LNU
◯ SCBU

What are the job positions of the people completing this survey?

☐ Senior Doctor (Consultant)
☐ Middle Grade Doctor (SpR)
☐ Junior Grade Doctor (SHO)
☐ Nurse
☐ Dietitian

What band of nurse are you?

____

What band of dietitian are you?

____

Does your unit have written feeding guidelines/ protocol for medical and surgical babies?

◯ Yes
◯ No

We would greatly appreciate it if you would upload the feeding guidelines/protocol using the 'upload document' link below. Alternatively, they can be emailed to the GASTRIC study team at gastric.study@liverpool.ac.uk.

Are your standard NG feeds:

◯ Intermittent
◯ Continuous

Is there specific guidance about how Gastric Residual Volume should be measured and interpreted - for example a protocol or guideline?

◯ Yes
◯ No

We would greatly appreciate it if you would upload the guidelines/protocol using the 'upload document' link below. Alternatively, they can be emailed to the GASTRIC study team at gastric.study@liverpool.ac.uk.

Does Gastric Residual Volume measurement differ between the medical and surgical babies?

◯ Yes
◯ No

Please describe how it differs?

The rest of the survey is now ONLY asking about the management of preterm and term medical babies - not surgical babies.

How often do staff in your unit measure Gastric Residual Volume? (this question is NOT about aspirating a small amount to test pH, ONLY about how often you measure Gastric Residual Volume)

- ☐ Once a day
- ☐ Before every feed
- ☐ Only when clinically indicated
- ☐ Other

Please provide further details regarding how staff measure Gastric Residual Volume below; if 'other' has been selected, please also describe below:

Are Gastric Residual Volume measured for all babies, or just below a set gestational age/birth weight or for a specific condition?

Is the specific guidance for Gastric Residual Volume measurement followed and actually undertaken as per protocol? If needed, please ask a clinical member of your nursing team.

- ◯ Always
- ◯ Usually
- ◯ Often
- ◯ Rarely
- ◯ Never

Why is the guidance not always followed?

Who usually decides what to do with concerning Gastric Residual Volume aspirates in the first instance?

- ☐ Senior Doctor (Consultant)
- ☐ Middle Grade Doctor (SpR)
- ☐ Junior Grade Doctor (SHO)
- ☐ Bedside Nurse
- ☐ Nurse in charge of shift (senior nurse)

What band of nurse?

What would make a nurse seek advice from the medical team?

If you indicated that more than one person is involved in decision-making, please explain which factors may influence who makes the decision below:

How much does volume of the aspirate affect your decision around Gastric Residual Volume?

◯ 1 (Not at all) ◯ 2 ◯ 3 ◯ 4 ◯ 5 (Very much)

Please specify how:

How much does colour of the aspirate affect your decision around Gastric Residual Volume?

◯ 1 (Not at all) ◯ 2 ◯ 3 ◯ 4 ◯ 5 (Very much)

Please specify how:

What do you do with Gastric Residual Volume once obtained: return it or discard it?

◯ Return
◯ Discard
◯ Other

Please explain the factors that influence the decision to return or discard the Gastric Residual Volume. If 'other' selected, please also explain below:

Thank you for participating in the survey, your responses are greatly appreciated! If you have any further comments you wish to make, please provide below:

If you wish to be contacted about the study, please add your email address below (optional):

____

Only

Supplementary Table 1: Individual responses to the question "Are Gastric Residual Volume measured for all babies, or just below a set gestational age/birth weight or for a specific condition"?

	Frequency
<32/40	2
All Babies	**30**
All babies on high risk feeding regimes	1
All babies until on full feeds	1
All babies weighing <1500g at birth who have not reached full feeds	1
All babies with NG tubes in until decision to stop monitoring ie for long term tube feeding.	1
All babies with NG tubes.	1
All babies with NGT/ OGT in-situ	1
All babies with tube feeding whilst the feeds are being increased	1
All tube fed babies	1
Any baby with a gastric tube in situ - but again, this is very variable depending on the nurse looking after the baby	1
As clinically indicated	1
Babies at high/moderate risk [of NEC] on our enteral feeding guideline - <32 weeks	1
Currently for all babies but stopping soon unless unwell.	1
For all babies on NG tube feeds	1
For premature babies or for sick babies on iv fluids and hourly feeds. NIC not aware of gestation cut off.	1
For specific conditions	1
GRV will be measured for babies who are on an increasing feed volume, to assess how well the baby is absorbing feed and to determine if the baby will be able to tolerate an increase in their milk volume.	1
GRVs are routinely measured 4-6 hourly for all babies	1
If clinically indicated. No set policy.	1
If requested by medical team	1
In intensive care - yes or in babies who are grading up to full enteral feeds	1
It would depend on the clinical situation	1
Measure for all babies when commencing enteral NGT feeds	1
NO	1
No baby with only medical issues; will depend on surgical condition/ stage pre and post surgery in surgical babies	1
No set criteria.	1
No set cut-offs, individual nurses make decision to measure or not	1
No. Only if showing signs/symptoms of not tolerating feeds.	1
No. babies within ICU may have GRV measured when establishing feed.	1
Not routinely done	1
Only if clinically indicated or if on continuous feeds (used in smallest babies below <34 weeks)	1
Only those being fed more frequently than 3 hourly - no specific cut-off but in general <32 weeks or IUGR or concern about antenatal Dopplers	1
Only when clinically indicated in any baby	1
Only when clinically indicated.	1
Our practice is not to empty out the stomach completely to measure residual volume. If a significant volume is aspirated prior to the next feed(~50% of the feed volume), then the feed is replaced unless there is concern regarding the nature of the aspirate or the clinical condition of the baby.	1

The nature of the aspirate is often commented on. In an observational study, green stained aspirates were of themselves not a marker of feed intolerance if of small volume (<2mL for infants <750g, or <3mL for infants <1kg). However, Advancing enteral feeds Weight <1001g 1001-1250g 1251-1500g or <33/40 Stage 2 Initial Nutritive Feeds 30ml/kg/day 30ml/kg/day 30ml/kg/day Stage 3 24 Hourly Increments 30ml/kg/day 30ml/kg/day 30ml/kg/day a dark stained gastric aspirate is generally accepted as abnormal when a feeding tube is believed to be correctly positioned in the stomach. An appropriate GRV is <25% of preceding volume administered since last replacement of gastric aspirate. In this case, the aspirated volume should be replaced in full. A volume of GRV >25% but less than 1.5mL is unlikely to be of concern and can again be replaced. A GRV of 25-50% of given volume is relatively high. If the infant is well and there is no clinical evidence of NEC, then the normal hourly volume should be replaced only. Feeds may be continued, but there should be no increments in feed until GRV has been below 25% on two consecutive occasions. A GRV of greater than 50% of preceding volume administered is excessive and should prompt a clinical assessment. If the examination is unremarkable and the infant is well, the hourly amount should be replaced and the feed omitted. If the following aspirate before the next scheduled feed has reduced then feeds can be continued, but sustained aspirates of >50% should prompt further review and withholding feeds for 12 to 24 hours even when clinical assessment is normal. On restarting feeds, it is recommended to recommence feeds at 10ml/kg/d for 6 hours, then 50% of previously attained feeds for 6 hours then back to previously attained feed rate. No further increments should be attempted for a further 24 hours. Lightly bile stained aspirates can be tolerated if of accept See attached feeding guideline as above	1
This is not stated precisely in our guidelines. Anecdotally the smaller more immature babies and the surgical babies get them measured more frequently	1
We always check the tube before a feed is given but we only measure GRV if indicated and this would be in all babies If when checking the tube the nurse finds it easy to withdraw milk then she will often check there isn't a large residual volume.	1
We would normally not measure gastric residual volume before ngt feeding a baby as used to be done in the past. A small amount to test pH is all that is done here.	1
Yes	1
all babies being NGT fed	1
all babies if ng tube in place	1
all babies on NGT feeds	1
all babies until fully fed once on full enteral feeds will just check pH	1
all tube fed	1
as above	1
measured for all as clinically indicated.	1
most/all	1
n/a	1
no	2
no set gestation (all NG fed babies)	1
no specific gestational age.	1
not routinely measured	1
only preterm babies or surgical patients following surgery	1
only sub 27 weeks and clinical concerns	1
only those ng fed	1
those below certain gestation/weight and if not on full feeds.	1
volume not routinely measured, unless keeping strict in/out record	1
	90

Supplementary Table 2: Detailed summary of UK NU enteral feeding written guidelines

	Neonatal unit level*	Default feeding method	GRV checking	Threshold for stopping feeds
**1.**	LNU	Bolus feeds with advancement strategy as per SIFT trial	Not specifically mentioned	Aspirate >50% feed volume or green aspirates
**2.**	NICU	Bolus feeds	Routinely measured but no mention of frequency or technique	Aspirate >50% feed volume in previous 6 hours or bilious aspirates
**3.**	NICU	Bolus feeds	Measured but no mention of frequency or how	Aspirates >50% or >1ml<br>If aspirate contains blood or bile.<br>Discard GRV, stop feeds, wait 2 hours and re-assess
**4.**	NICU	Bolus	No mention of frequency or technique	Consider stopping if<br>● pre-feed aspirate >4mls/kg,<br>● heavy bile stained aspirates or<br>● 2 vomits after consecutive feeds
**5.**	LNU	Bolus with advancement as per SIFT trial	GRV aspirated 4 hourly	GRV >25% feeds in previous 4 hours combined with abdominal distention and/or vomiting
**6.**	NICU	No mention	No mention	No mention
**7.**	NICU	Bolus feeds and advanced as per SIFT trial	Check GRV no more than 6 hourly unless concerns	Withhold feeds for 6-12 hours if GRV >40% of feed given or 2 or 3ml (dependant on infant weight), heavily bile or blood stained or abdominal distention
**8.**	LNU	Not stated	Not stated	4 hourly NG aspirates are <25% of total infused in the preceding 4 hours<br>No significant abdominal distension<br>No significant vomiting<br>No bile -<br>stained aspirates
**9.**	NICU	Bolus feeds	Not stated	GRV >50% volume of feeds over last 6 hours or vomit of this size
**10.**	NICU	2 hourly bolus advanced as per SIFT	4-6 hourly	*Action with gastric residuals:*<br>If aspirates 25-50% of total, replace the volume, omit the feed and do not increment.<br>If aspirates >50% of total, stop feeds and medical review.<br>If dark bilious rather than lightly bile stained, stop feeds and medical review
**11.**	NICU	Bolus feeds, 2 risk levels, advanced as per SIFT	Routine measurement of full gastric residuals should be avoided. This should only be done, with discussion, as a part of<br>a full medical/ ANNP	Signs of feed intolerance may include clinical observations such as desaturation and bradycardia events and increased work of breathing, vomiting, abdominal distention and discolouration.
**12.**	SCU	Bolus feeds	Not specified	GRV > 2 hourly amount, vomiting or abdominal distention
**13.**	NICU	Bolus feeds	Not specified	**Medical babies:**<br>2ml/kg of milky gastric residual is not important and should simply be replaced. Where the<br>gastric residual at higher volumes is equivalent to 100% of the bolus, then the feeds should be<br>stopped and a clinical review<br>**Surgical babies:**

				● aspirate <½ feed volume since last aspirate replace the aspirate itself and continue feeding<br>● aspirate ≥ ½ feed volume but <whole feed volume replace half of the aspirate and discard the rest<br>● aspirate ≥whole feed volume since previous aspirate do not replace the aspirate, stop feeding & obtain senior medical and surgical review
**14.**	NICU	Bolus feeds	4 - 6 hourly	Examine and assess the baby if<br>● vomiting,<br>● GRV s >25% of the previous 4 hours total feed volume<br>● residuals are persisting or increasing<br>Small milky / yellow aspirates up to 2-3 mls are frequently normal. They can be replaced, and feeds continued
**15.**	LNU	Bolus feeds	Not specified	Aspirates up to 2-3ml or 50% of the previous 4 hours feed can be normal if the baby is well<br>Aspirates greater than 50% of the previous 4 hours feed or 2-3ml (whichever is greater) discard aspirate, hold feed and try again in 2 hours<br>If aspirate contains blood or bile then stop feeds
**16.**	NICU	Bolus feeds	4 - 6 h	When babies are on any enteral feeds, only aspirate the stomach contents via<br>a gastric tube every 4 - 6 hours, in order to check the residual volume. The assessment of the baby should include any abdominal distension, dark green (bilious) aspirates and bowel opening,<br><br>If <50% of the previous 4 - 6 hour total feed volume is aspirated, then replace the aspirate and continue enteral feeding, provided the baby is<br>otherwise clinically stable<br><br>If >50% of the previous 4 - 6 hour total feed volume is aspirated, then discuss with medical staff; often reasonable to replace the aspirate and omit the feed. If necessary, stop the feeds for 4 - 6 hours; a senior member of the medical / nursing team should then review
**17.**	NICU	Bolus feeds	Q 6 until infant is fully fed	Signs of intolerance<br>1. Vomiting<br>2. Gastric residuals >25% of previous 6<br>hours feed volume, persistent or increasing<br>3. Abdominal distension/increasing abdominal girth<br>4. Increase in stool frequency
**18.**	LNU	Bolus feeds	Not specified	If the aspirates are non-bilious and less than half the volume of previous feed they can be replaced and feeding continued while observing the infant closely<br><br>If the aspirates are bilious or >50% of the previous feed volume, consider withholding the feeds on that occasion and assess for any signs of NEC
**19.**	NICU	Bolus feeds	Not specified	Large volume aspirates or dark green bile stained aspirates, particularly in association with abdominal distension and/or tenderness are a cause for concern. Small milky / yellow aspirates up to 2-3 mls are frequently normal. They can be replaced, and feeds continued
**20.**	LNU	Bolus feeds	Not specified	No mention
**21.**	NICU	Bolus feeds	No more than 4 - 6h	If vomit or GRV exceed 33% of the last feed volume or are more than 3.5 mls in a single aspirate then examine baby<br>Small residuals normal

**22.**	NICU	Bolus feeds	Not specified	Isolated large GRV in the absence of other clinical signs & symptoms should not prevent continued feeding<br>Signs of intolerance:<br>● Vomiting<br>● GRV >30% of previous 5 hours feed<br>● Abdo distention<br>● Unwell baby
**23.**	LNU	Bolus	4-6 hourly	If GRV 25-50% of total, replace the hourly amount, omit the feed and do not increase<br>If GRV >50% of total, stop feeds and medical review
**A.**	Network	Bolus	Not specified	GRV >25% (some >50%) in previous 4 hours in combination with vomiting and abdominal distention + bilious aspirates
**B.**	Network	Bolus feeds	4 hourly	Stop feeds if GRV heavily blood or bile stained<br>No mention of volume
**C.**	Network	Bolus feeds	Not specified	GRV should not be used in isolation to determine feed tolerance<br>Intolerance:<br>Vomiting + GRV >50% in the last 4 hours (especially if increasing) + abdominal distention
**D.**	Network	Bolus feeds advanced as per SIFT	Not specified	Infants 'feed tolerance' assessed with each set of cares (high risk), assess twice daily (mod risk) and before making changes in feed volumes (standard risk)<br>Assessing tolerance:<br>Undigested gastric residuals using a colour chart<br>GRV not used in isolation<br>But vomiting, GRV >25% of feed volume in last 4 hours + bloody or bilious residuals + abdominal distention
**E**	Network	Bolus	Assess GRV 4 - 6 hourly depending on cares	If GRV >50% of total, stop feeds and medical review<br>If GRV 25-50% of total, replace the hourly amount, omit the feed and do not increase<br>An appropriate GRV is <25% of preceding volume since last replacement of GRV<br>Replace GRV in full<br>A GRV >25% but <1.5mls unlikely to be problematic<br>A GRV of 25-50% is high, but acceptable if well, replace only normal hourly volume and continue feeds but do not increase<br>A GRV >50% is excessive, perform clinical exam, if acceptable hourly volume can be replaced but feed withheld

*Neonatal Unit Level determined by NNAP 2017 report (https://www.hqip.org.uk/resource/national-neonatal-audit-programme-2017-annual-report-on-2016-data/)

Abbreviations: GRV = Gastric Residual Volume; SIFT = Speed of Increasing of milk Feeds Trial(23), NICU = Neonatal Intensive Care Unit, LNU = Local Neonatal Unit, SCU = Special Care Unit.
